# Supplementary material for: Unsupervised Gait Event Identification with a Single Wearable Accelerometer and/or Gyroscope: A Comparison of Methods across Running Speeds, Surfaces, and Foot Strike Patterns
Source: Sensors (Basel). 2023 May 24;23(11):5022. doi: 10.3390/s23115022 (PMC10255839; doi:10.3390/s23115022)
Supplement: Supplementary file 1 [file sensors-23-05022-s001.zip › sensors-2408566-supplementary.pdf]

Supplemental materials

Overview

Here we provide details for both our own IMU data processing and for each of the gait event identification methods included in our main paper. For details beyond what we provide here please refer to the original sources. Figures have been included showing the process each method uses to identify initial contact (IC) and terminal contact (TC) gait events. All data for these figures was taken from the same randomly selected trial (a 1.64 m tall 62.37 kg female running 3.73 m/s on the 'floor' surface and contacting the force plate with a forefoot strike angle of -0.12 rad). Wherever possible, figures originally published with each method were digitized (https://apps.automeris.io/wpd/) and run through the code provided with this paper to ensure we could faithfully reproduce results (not included here due to copyright). For clarity, we have standardized the original coordinate conventions used in each method to be consistent with the segment- and wearable-coordinate systems (SCS and WCS) described briefly in our main paper (Fig. 3), described more fully in our IMU processing Supplement (below), and with ISB recommendations [50]. Finally, each method's performance is individually reported here. Please note that figure axes and descriptions are consistent across all methods; consequently, the results for some methods may not be fully plotted if they exceed the axis range. Also note, the results figures are only fully described the first time they appear (Figure S2).

Timing Constraints

For several methods, novel timing constraints were added to facilitate pattern recognition and exclude observations falling outside the values reported for steady-state running [1-9]. Based on these sources, we adopted a maximum step frequency of 4.75 Hz, stance times between 95 and 270 ms, and swing times between 200 and 600 ms.

Common output

Finally, for all methods, to prevent errors (e.g., empty IC and TC outputs, impossible patterns of results such as IC-IC-TC-IC, etc.) that were common to many methods (all except: Mizrahi, Benson, Norris, Reenalda, and Whelan) and to ensure a common output, we implemented a function that took the raw IC, TC, and side output from each function and checked it to ensure: (1) gait events were identified (if a method was supposed to deliver both ICs and TCs, only paired gait events were accepted; i.e., no IC without TC), (2) gait event timings were unique and in temporal order, and (3) gait events followed a logical IC-TC-IC-TC pattern.

Shank-mounted wearable methods

Mizrahi method

Developing and validating a gait-event identification method was not the primary focus of Mizrahi et al. [10]. The authors did, however, report identifying IC from the peak acceleration of a uniaxial accelerometer on the tibial tuberosity aligned with the longitudinal axis of the tibia ($a_{WCS,y}$). The authors reported using an automated code that we attempted to replicate based on their paper. Our code finds maxima in y-axis acceleration (proximal-distal in the SCS or ~longitudinal in the WCS) with a minimum separation of ~422 ms (based on the maximum step frequencies reported across previous studies) (Fig. S1). For results see Fig. S2.

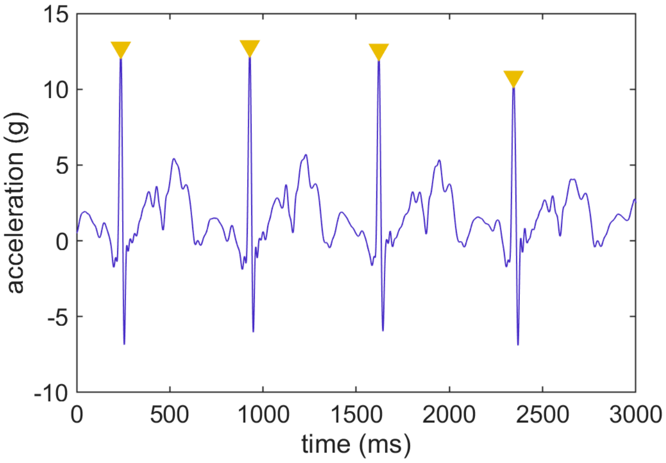

Figure S1. *Y*-axis acceleration (proximal-distal in the SCS; ~longitudinal in the WCS; dark blue line) maxima are found with a minimum separation of ~422 ms and selected as IC gait events (gold triangles). No TC identification.

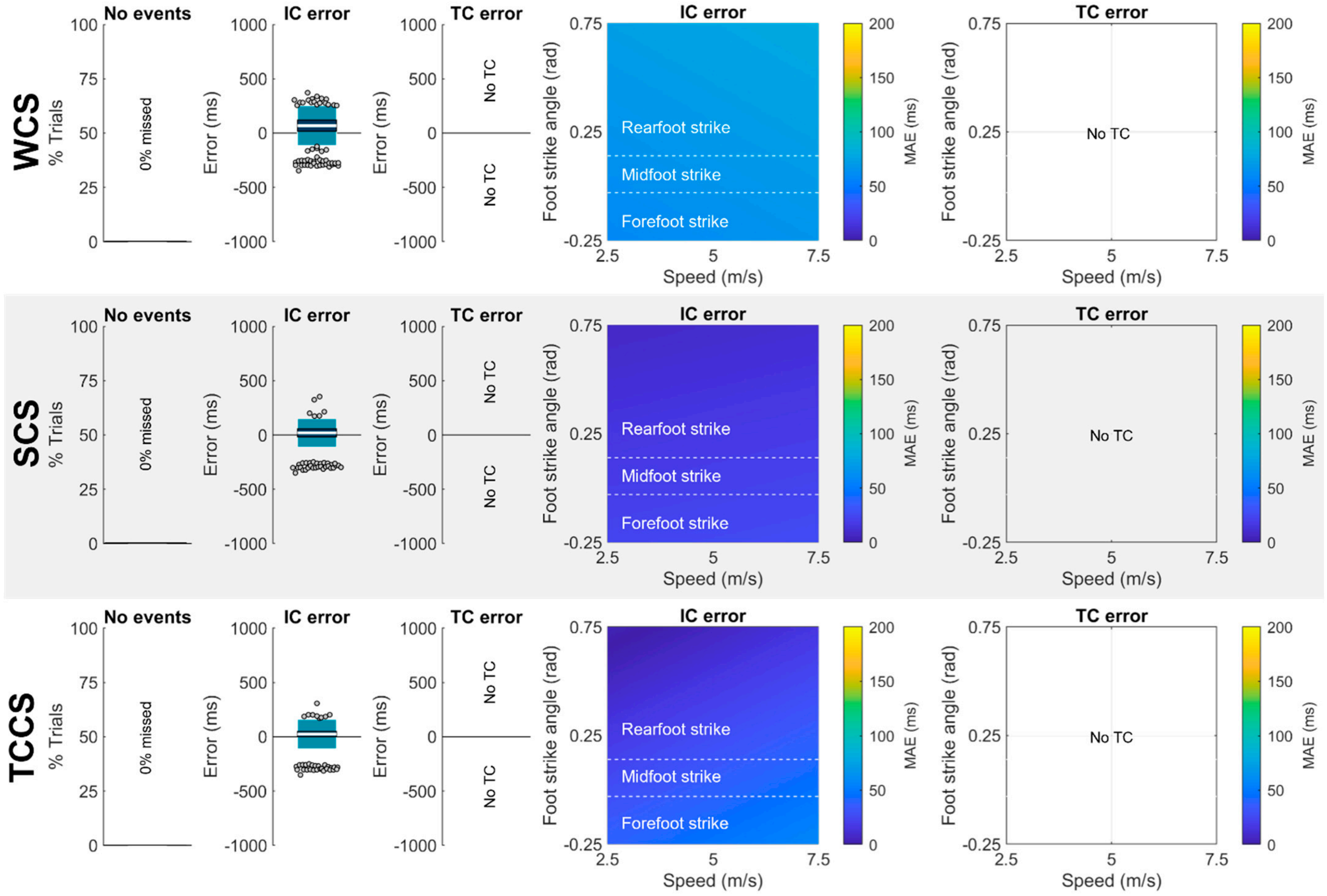

Figure S2. Results were calculated using the methods described in each coordinate system (calculated as described in part 8 of our IMU processing breakdown at the end of this Supplement). Results in the **top row** are for the wearable coordinate system (WCS), results in the **middle row** are for the segment coordinate system (SCS), and results in the **bottom row** are for a pseudo-global coordinate system, the "tilt-corrected" coordinate system (TCCS). Reading left to right, in the **first column** the white horizontal line represents the mean percentage of trials per participant without any gait events recognized, dark blue represents ±95% confidence interval (±1.96 SEM) around the mean, light blue represents ±1 SD around the mean. Gray dots represent participants outside ±1 SD. No bars indicates that gait events were identified in every trial for every participant. In the **second column** IC error means (white bar), ±1 within-method SD (dark blue), and ±95% LOA (1.96 SD of errors;

light blue) are plotted. Gray dots represent trials falling outside the 95% LOA. A value of 0 indicates perfect agreement with the ground truth. Positive values indicate the IC was estimated later than the ground truth (after the force plate IC). Negative values indicate the IC was estimated earlier than the ground truth (before the force plate IC). In the **third column** TC error means (white bar), ±1 within-method SD (dark blue), and ±95% LOA (1.96 SD of errors; light blue) are plotted. Gray dots represent trials falling outside the 95% LOA. A value of 0 indicates perfect agreement with the ground truth. Positive values indicate the TC was estimated later than the ground truth (after the force plate TC). Negative values indicate the TC was estimated earlier than the ground truth (before the force plate TC). In the **fourth column** mean absolute error in IC predicted by a mixed effects model. Plotted as a function of speed and foot strike angle. Darker blue values represent lower predicted mean absolute errors while brighter yellow values represent higher predicted mean absolute errors. In the **fifth column** mean absolute error in TC predicted by a mixed effects model. Plotted as a function of speed and foot strike angle. Darker blue values represent lower predicted mean absolute errors while brighter yellow values represent higher predicted mean absolute errors.

Mercer method

Developing and validating a gait-event identification method was not the primary focus of Mercer et al. [11], however, they provided the first published details on a gait-event identification method previously used by Shorten and Winslow [12], Hamill et al. [13], and Derrick et al. [14, 15]. We attempted to translate these details into an automated code. To identify IC, the authors reported placing an accelerometer on the anteromedial distal tibia and finding a local minimum immediately preceding a global maximum in $a_{WCS,y}$ (Fig. S3A-C). To identify TC, they reported finding the "minimum after a second local maximum." We found that small local maxima could generate results inconsistent with the figures presented in Mercer's paper when looking for local maxima to identify TCs (indeed, their figure depicts the TC after *three* small local maxima). Thus, to exclude smaller local maxima, we added an additional constraint that only the four largest maxima in the window of interest would be considered. Results are shown in Fig. S4.

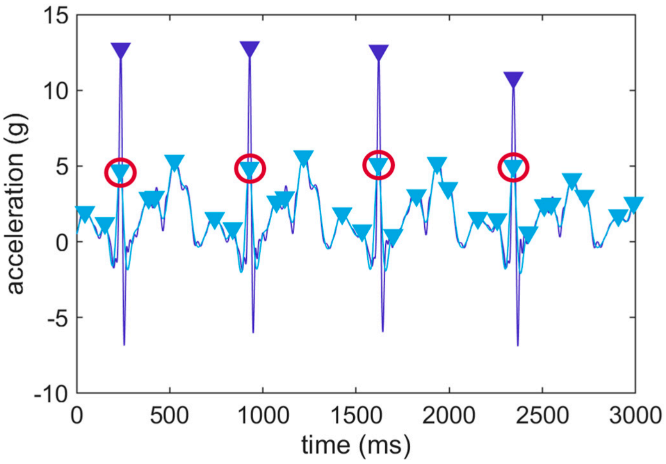

Figure S3: **(A)** Y-axis acceleration (proximal-distal in the SCS or ~longitudinal in the WCS; dark blue line) maxima (dark blue triangles) are found with a minimum separation of ~422 ms. The signal is then low-pass filtered at 15 Hz (light blue line) and maxima are found in the filtered signal with no minimum separation criterion (light blue triangles). The peak in the filtered signal closest to the peak in the original signal is selected as a peak of interest (red circle).

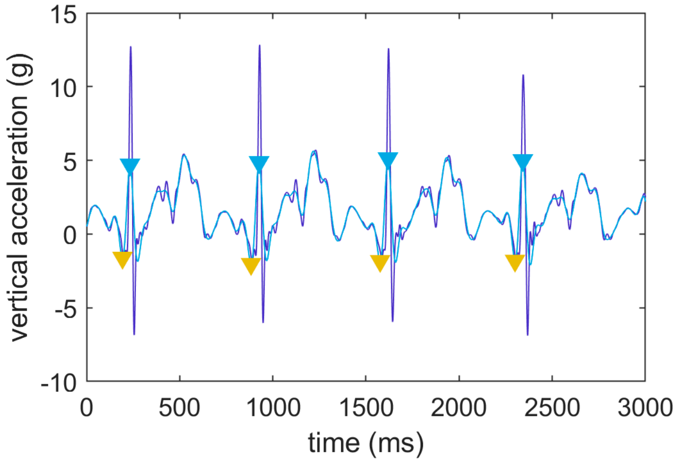

(B) Walking back from each peak of interest (light blue triangles) the first point when the signal magnitude begins to increase is selected as an IC gait event (gold triangles)

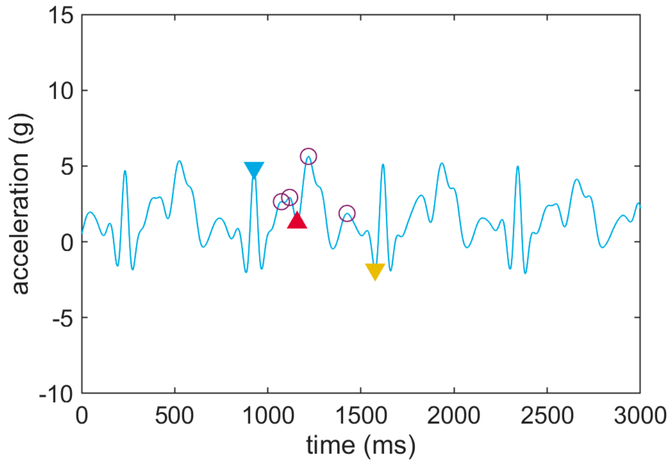

(C) The four largest maxima (red circles) are found between each peak of interest (light blue triangle) and its proceeding IC (gold triangle). Stepping forward from the second maxima, the first point when the signal magnitude begins to increase is selected as a TC gait event (red triangle).

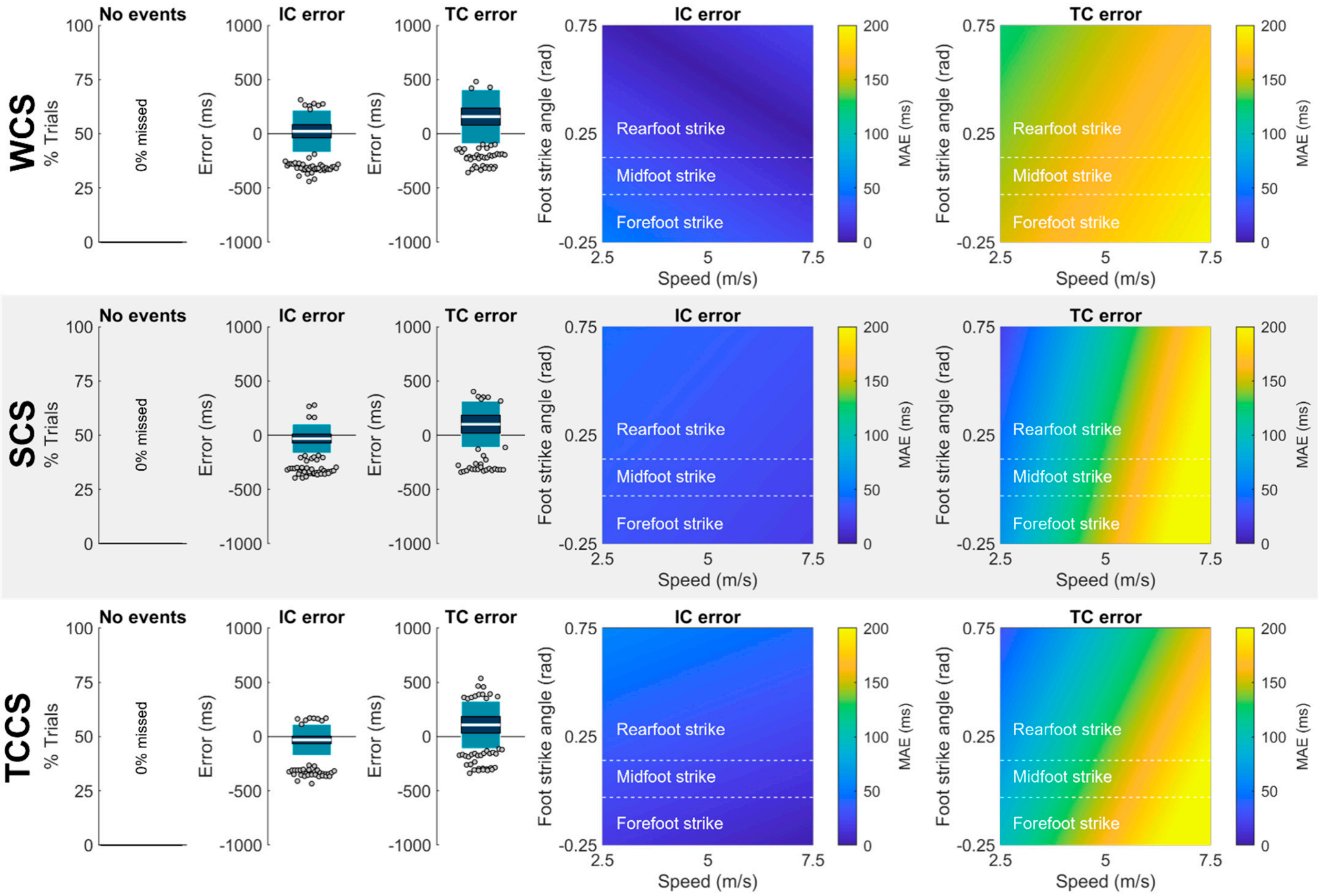

Figure S4. See Figure S2 for description.

Purcell method

Purcell et al. [16] placed a tri-axial accelerometer on the anteromedial tibia. To define IC, the authors report using minima in $a_{WCS,x}$ (as defined by the coordinate convention they report standardized to ours) that corresponds in time with a maxima in $a_{WCS,res}$. However, the data they present (and the method which we were successfully able to reproduce) suggest that they misreported their coordinates and actually used minima in $a_{WCS,x}$ (rather than maxima). To define TC, they take the average time stamp of local minima in $a_{WCS,x}$ and $a_{WCS,z}$ (after correcting their coordinate system) (Fig. S5A-B). The $a_{WCS,z}$ minima were not very pronounced; thus, to aid performance, we imposed a further constraint here that the window to search for these local minima should be between 20-60% of one IC to the next. Results are shown in Fig. S6.

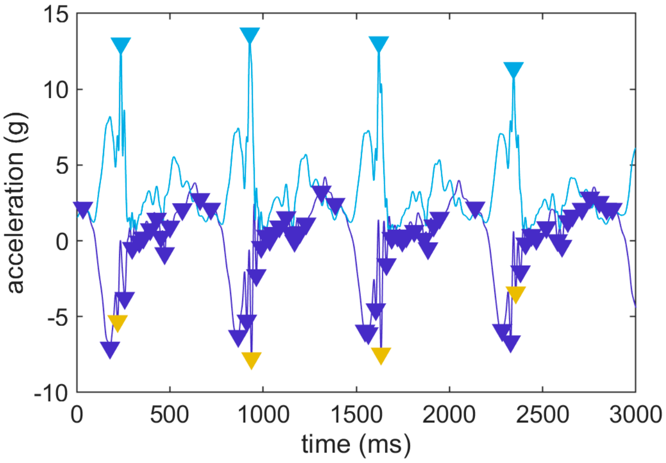

Figure S5: **(A)** *X*-axis acceleration (anterior-posterior in SCS, ~direction of progression in WCS; dark blue line) minima (dark blue triangles) are identified. Then, the resultant acceleration is calculated (light blue line) and peaks separated by at least ~422 ms are identified (light blue triangles). IC is defined as the *x*-axis minima closest to resultant maxima (gold triangle).

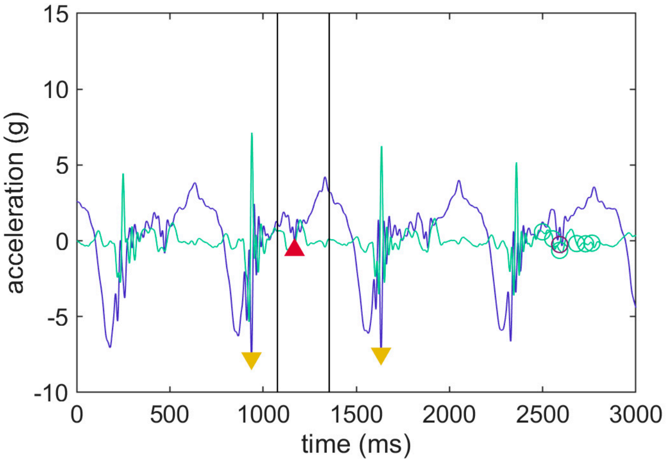

(B) A window of interest (vertical black lines) is created from 20-60% of one IC to the proceeding IC (gold triangles). The greatest magnitude *x*-axis minima in that window (blue circle) is found. All *z*-axis (medial-lateral in SCS, ~right-left in WCS) minima within that window (green circles) are found. The *z*-axis minimum closest to the large *x*-axis minimum (purple circle) is found. TC is defined as the midpoint between those two points (red triangle).

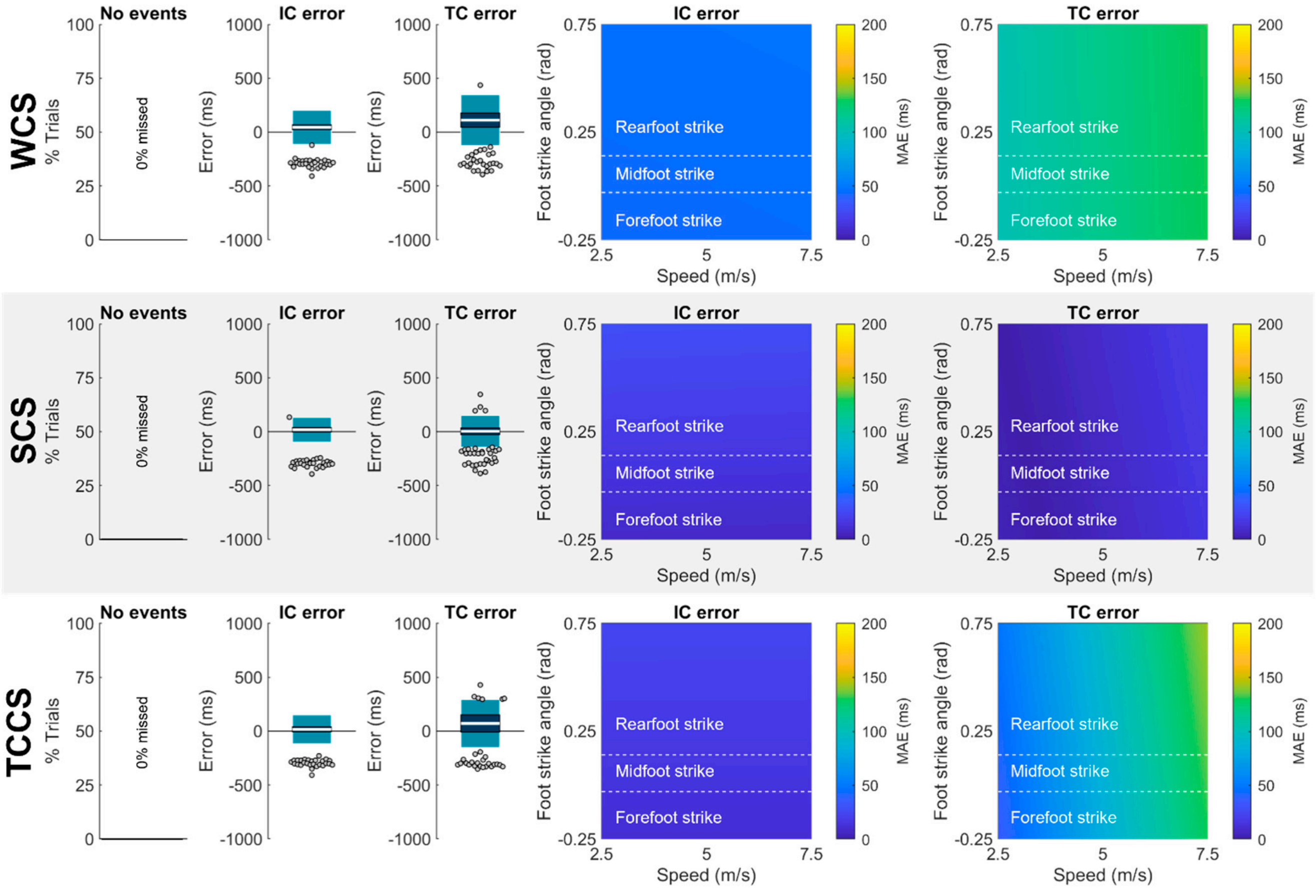

Figure S6: See Figure S2 for description.

Aminian/O'Donovan method

Aminian et al. [17] developed a method to identify gait events during walking that was later applied to running by O'Donovan et al. [18]. This method decomposes $\omega_{SCS,z}$ using a series of wavelet multi-resolution analyses (MRA) that split the signal into high-scale '*approximation*' (low-frequency) and low-scale '*detail*' (high-frequency) components. Aminian et al. report using two iterative 10-level 5th order Coiflet wavelet analyses. Given each level in MRA represents a frequency band that is a function of the sampling frequency used during data collection, we first resample data to match Aminian et al.'s reported 200 Hz sampling frequency (thus, yielding frequency bands that should be identical to theirs for each level; note, however, that Aminian et al. state their approximation "only considers" up to 36 Hz—consistent with a 72 Hz sampling frequency and not the reported 200 Hz sampling frequency—it is unclear where this discrepancy originates from but for the purposes of this paper we adhered to their stated sampling frequency and equations). After resampling, MRA is used to obtain a new signal approximation ("s_a") by summing details 1:9. Then, a second MRA is conducted on s_a and two new signal approximations are created: (1) $A_{2^1}s_a - A_{2^9}s_a$ is designed to enhance the IC component by subtracting approximation 9 from approximation 1 and (2) $A_{2^3}s_a - A_{2^9}s_a$ is designed to enhance the TC component by subtracting approximation 9 from approximation 3. These new signal approximations are then searched for maxima/minima within specific time constraints. Results shown in Fig. S8.

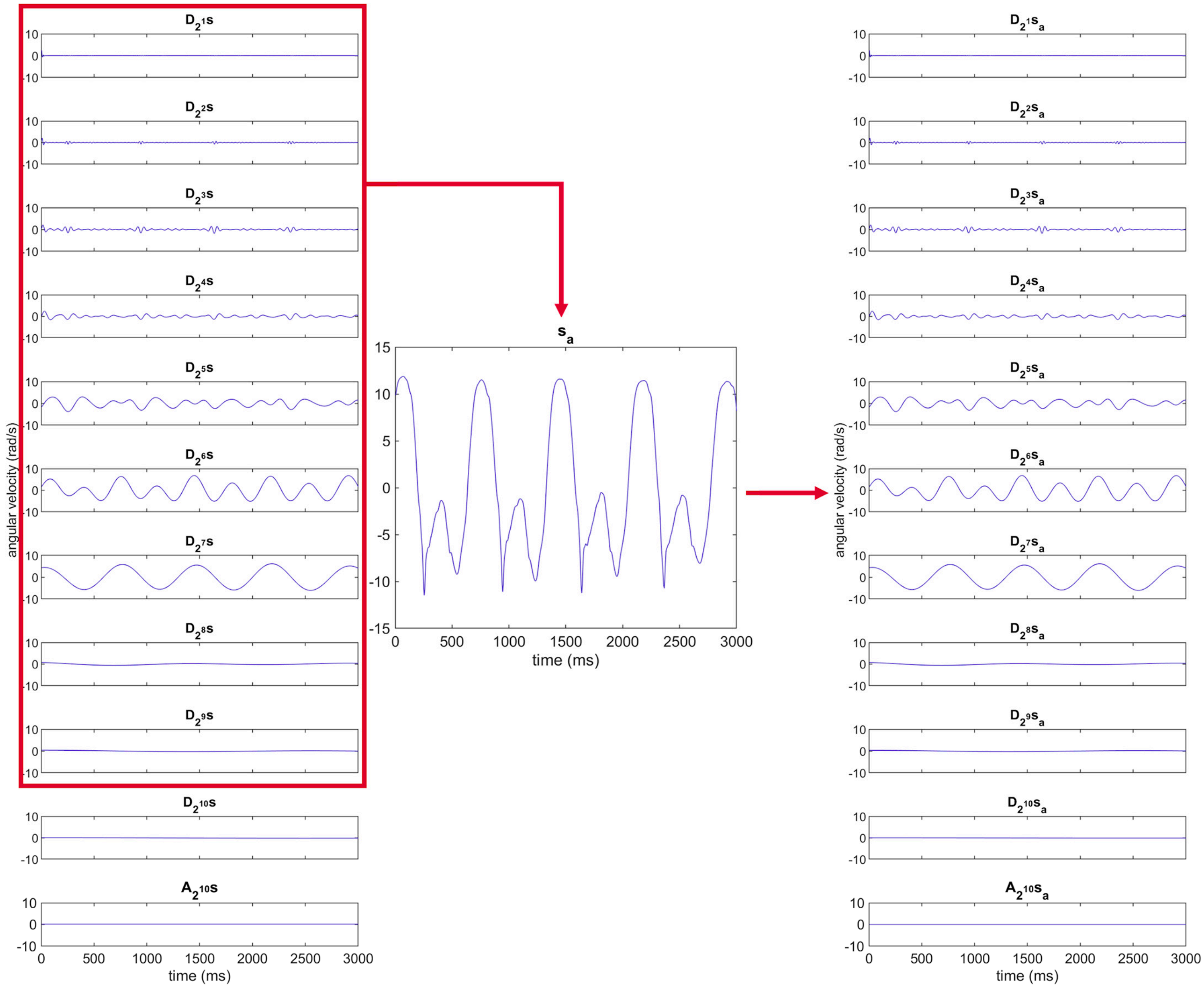

Figure S7. Angular velocity about the *z*-axis (medial-lateral in the SCS, ~right in the WCS) is resampled to 200 Hz then decomposed with a 10-level 5th order Coiflet wavelet multi-resolution analysis (MRA) **(A)**. Details 1 through 9 are summed to yield signal approximation s_a **(B)**. s_a is then entered into a second 10-level 5th order Coiflet wavelet MRA **(C)**.

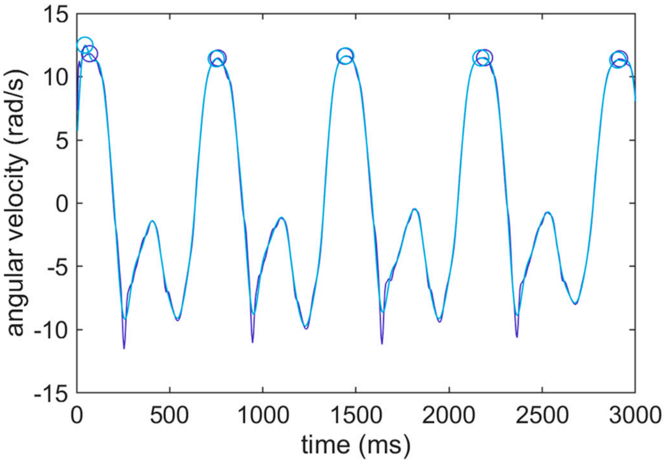

Figure S7: **(D)** Two new approximations are created: $A_{2^1}s_a - A_{2^9}s_a$ (dark blue line) and $A_{2^3}s_a - A_{2^9}s_a$ (light blue line). Maxima separated by at least ~422 ms are found in each signal approximation (circles). These maxima approximately correspond to mid-swing.

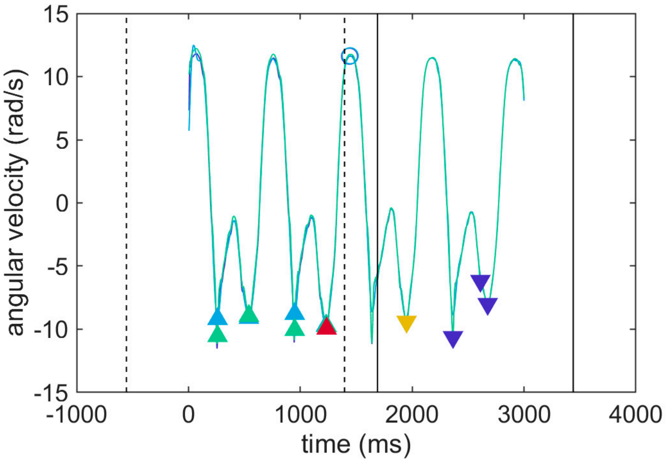

(E) A window of interest proceeding each maxima in $A_{2^1}s_a - A_{2^9}s_a$ (+250 to +2000 ms; solid vertical lines) is used to look for local minima corresponding to potential ICs (dark blue triangles). A window of interest preceding each maxima in $A_{2^3}s_a - A_{2^9}s_a$ (-2000 to -50 ms; dashed vertical lines) is used to look for local minima (light blue triangles) then the minimum values in the original signal s (green line) within 0 to +75 ms are selected as potential TCs (green triangles). Starting with the IC closest to the mid-swing maxima in time iterate backwards in time through potential TCs. The first potential IC-potential TC pair that satisfies the condition 100 ms < (IC - TC) < 2500 ms is accepted. If no pairing meets this condition iterate forward in time to the next potential IC. Note, the windows of interest stipulated by Arminian et al. for walking analysis were so large that they required the x-scale to be adjusted and extend beyond the data selected for analysis.

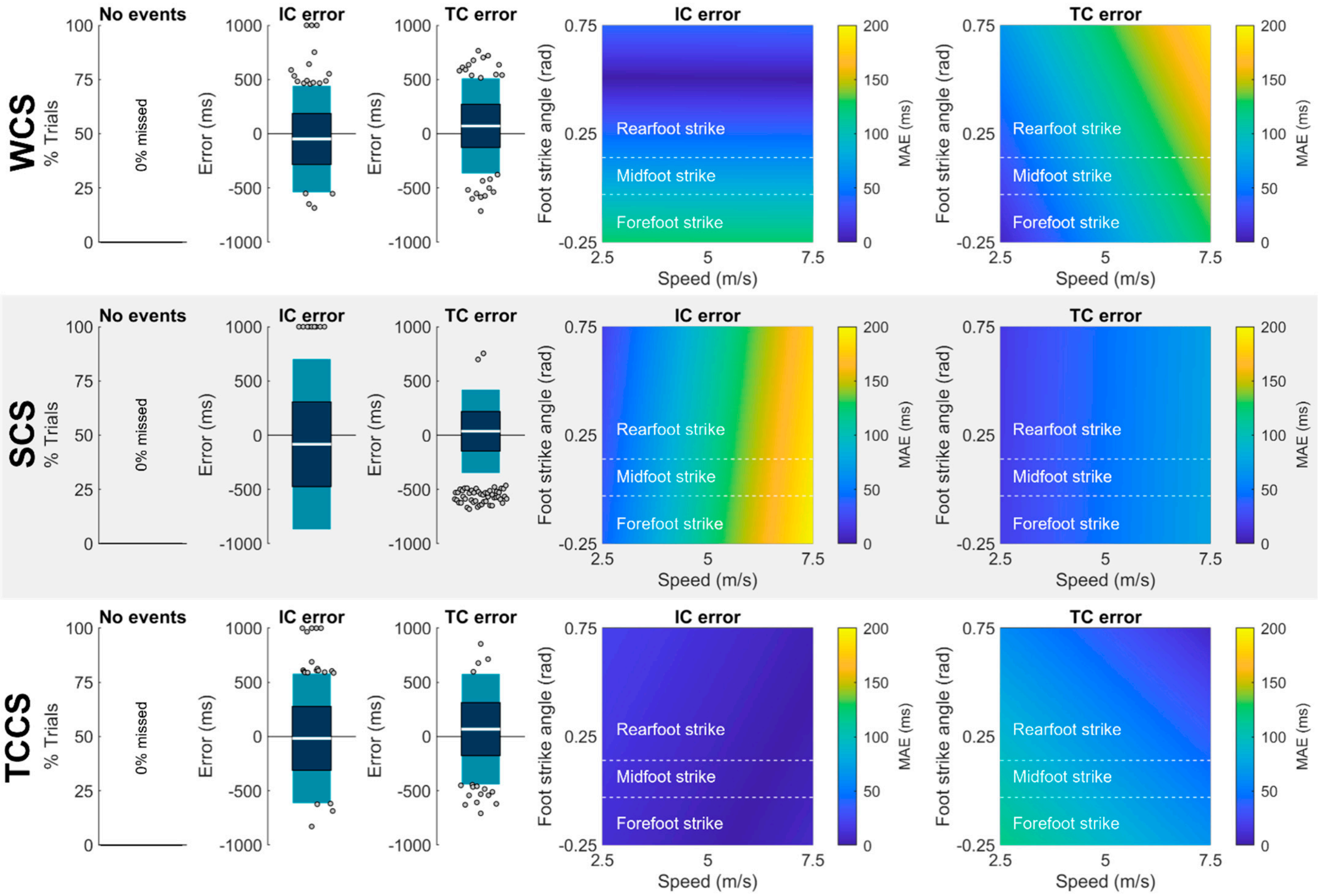

Figure S8: See Figure S2 for description.

Aminian/O'Donovan modified method

As stated in the previous section, Aminian et al. [17] developed their method to identify gait events in walking. O'Donovan et al. [18] later applied this method to running but did not report any adjustments to the windows of interest based on the temporal differences between walking and running. Here we adjust the windows of interest to better correspond to swing times presented in the running literature. The approach is otherwise as described above. Results shown in Fig. S10.

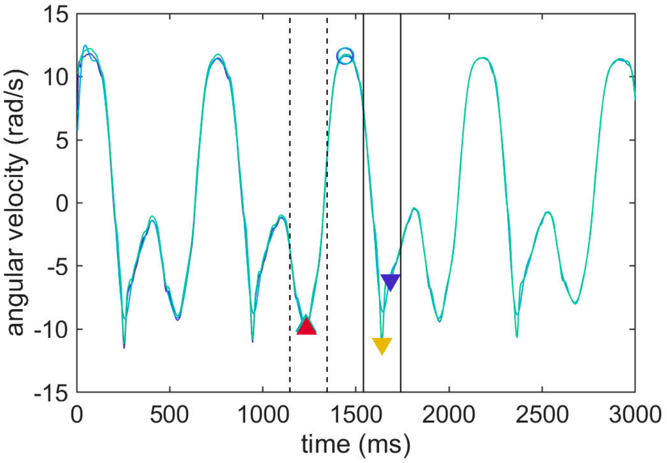

Figure S9: New running-based windows of interest are defined for the Amanian/O'Donovan approach. A window of interest proceeding each maxima in $A_{2^1}s_a - A_{2^9}s_a$ (+100 to +300 ms; solid vertical lines) is used to look for local minima corresponding to potential ICs (dark blue triangles). A window of interest preceding each maxima in $A_{2^3}s_a - A_{2^9}s_a$ (-300 to -100 ms; dashed vertical lines) is used to look for local minima (light blue triangles) then the minimum values in the original signal s (green line) within 0 to +75 ms are selected as potential TCs (green triangles). Starting with the IC closest to the mid-swing maxima in time iterate backwards in time through potential TCs. The first potential IC-potential TC pair that satisfies the condition 200 ms < (IC - TC) < 600 ms is accepted. If no pairing meets this condition iterate forward in time to the next potential IC.

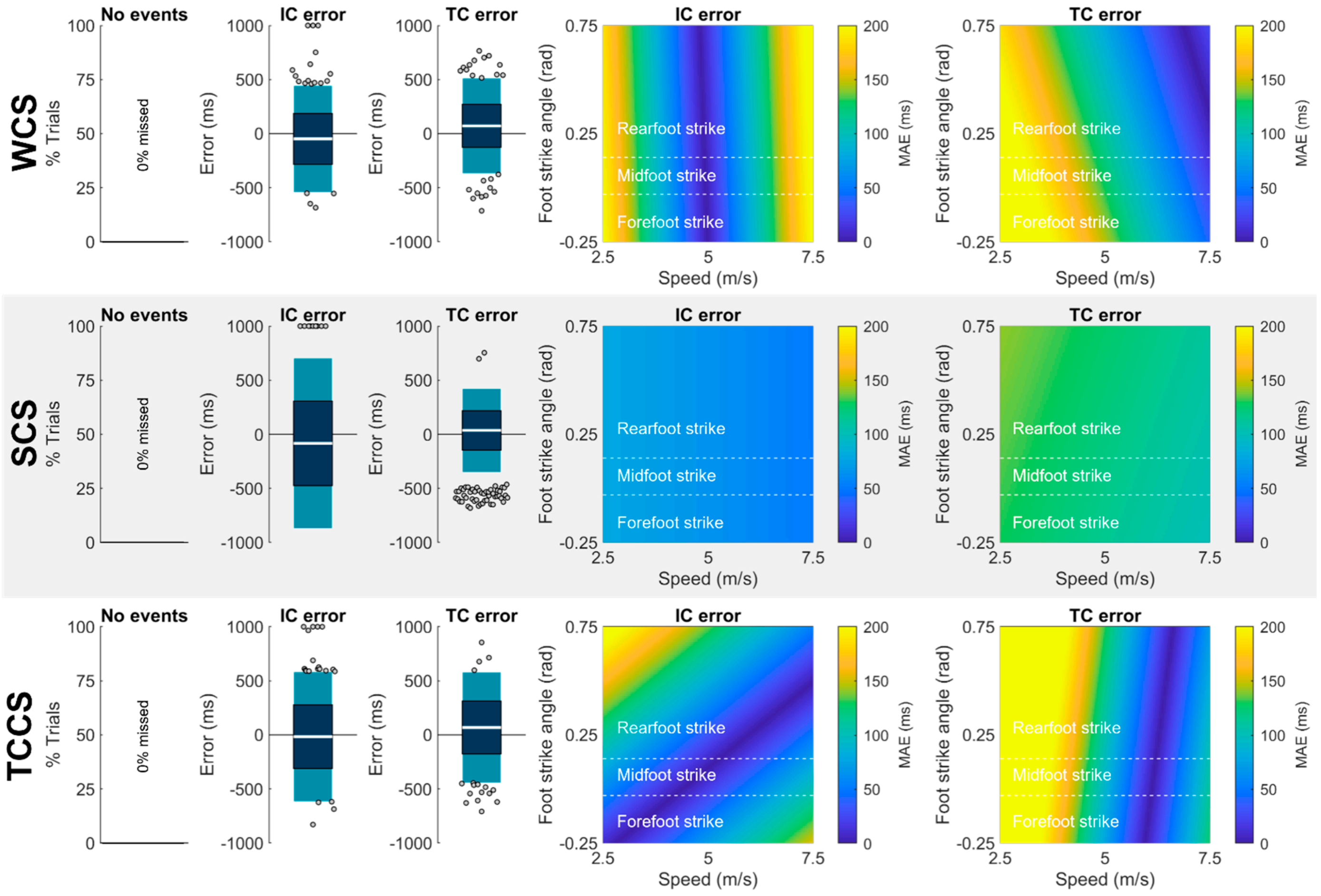

Figure S10. See Figure S2 for description.

Greene/McGrath method

Greene et al. [19] developed a method for gait event identification during walking that was later adapted by McGrath et al. [20] to identify running gait events. McGrath et al. placed IMUs mid-shank to capture $\omega_{WCS,z}$. Data were low-pass filtered with a 5 Hz 5th order Butterworth filter then thresholds calculated based on the data's properties were used to determine IC and TC. Finally, the sequence and temporal spacing of gait events

was checked to ensure they were logical (i.e., must follow IC-TC-IC-TC pattern). Results are shown in Fig. S12.

$$th_1 = 0.6 * max(\omega_{WCS,z})$$

$$th_2 = 0.8 * \frac{1}{N}\sum_{i=1}^{N}(\omega_{WCS,z,i} > \underline{\omega}_{WCS,z})$$

$$th_3 = 0.8 * \left|\frac{1}{N}\sum_{i=1}^{N}(\omega_{WCS,z,i} < \underline{\omega}_{WCS,z})\right|$$

$$th_4 = 0.8 * \frac{1}{N}\sum_{i=1}^{N}(\omega_{WCS,z,i} < \underline{\omega}_{WCS,z})$$

$$th_5 = \overline{\omega}_{WCS,z}$$

$$th_6 = 2 * th3$$

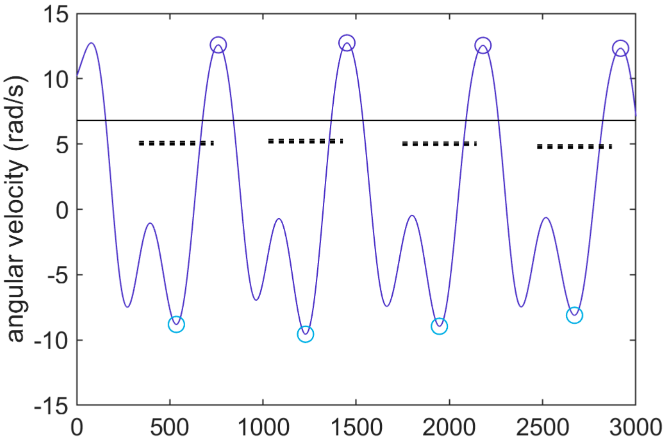

Figure S11: (A) Angular velocity about the *z*-axis (medial-lateral in the SCS, ~right in the WCS; dark blue line) is filtered with a 5-Hz 5th order low-pass Butterworth filter. Maxima separated by at least t_1 (500 ms) are identified (dark blue circles). Any identified maximum without a preceding minimum (light blue circles) at least th_1 below the maximum (dashed lines) or with a value below th_2 (solid line) is discarded.

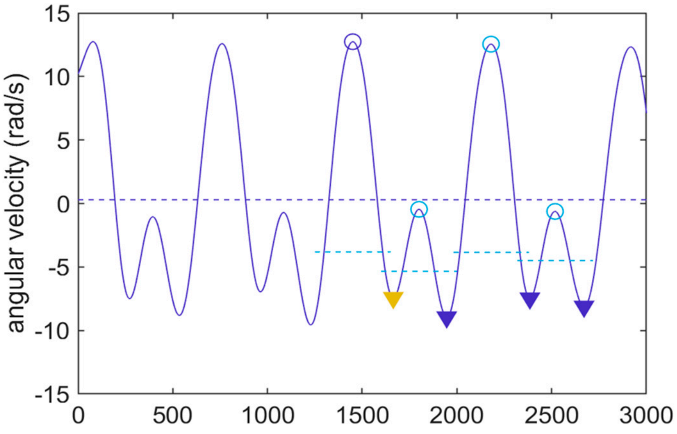

(B) Iterating backwards through the remaining maxima (dark blue circle) proceeding minima are identified as potential ICs (dark blue triangle). Potential ICs are rejected if their preceding maxima (light blue circle) is not at least th_3 greater than their magnitude (dashed light blue line) or if their magnitude is th_5 or greater (dashed dark blue line). The potential IC satisfying these conditions and closest in time to the maxima is labeled as the IC.

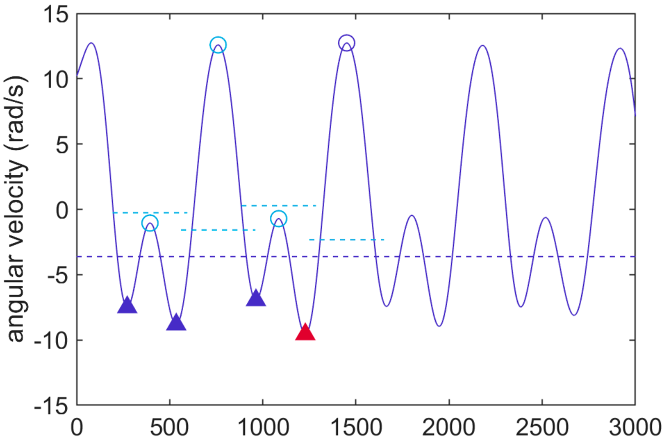

(C) Similarly, minima preceding the maxima are identified as potential TCs (dark blue triangles). Potential TCs are rejected if their magnitudes are th_4 or greater (dashed dark blue line) or their proceeding maxima (light blue circle) is not th_6 greater than the potential TC (dashed light blue line; note: Greene et al. stipulate this threshold applies to the ***pre***ceding maximum; however, we found that maxima preceding TCs rarely meet this condition—including on Greene et al.'s own data digitized via https://apps.automeris.io/wpd/—thus, we believe this was a typo and that this condition is intended to be applied to ***pro***ceeding maxima). The timing and sequence of events is then checked to ensure events always alternate between IC and TC, ICs and their proceeding TCs occur within 2500 ms of each other, and TCs and their proceeding ICs occur within 7500 ms of each other.

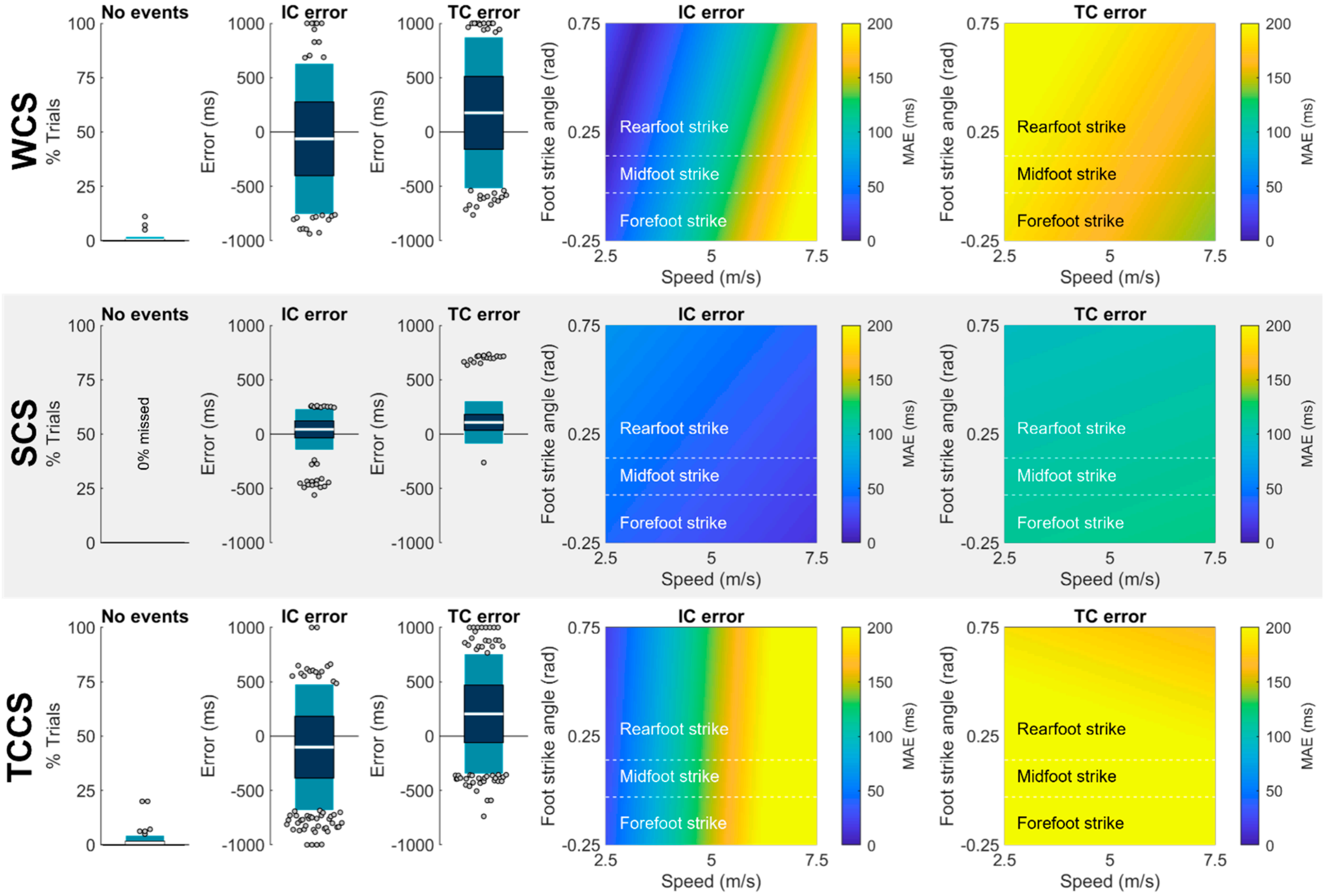

Figure S12. See Figure S2 for description.

Greene/McGrath modified method

As stated in the previous section, Greene et al. [19] developed their method to identify gait events in walking. McGrath et al. [20] later applied this method to running but did not report any adjustments to the algorithm based on the temporal differences between walking and running. Here we adjust the algorithm to better correspond to the

timing between gait events presented in the running literature. The approach is otherwise as described above. Results are shown in Fig. S13.

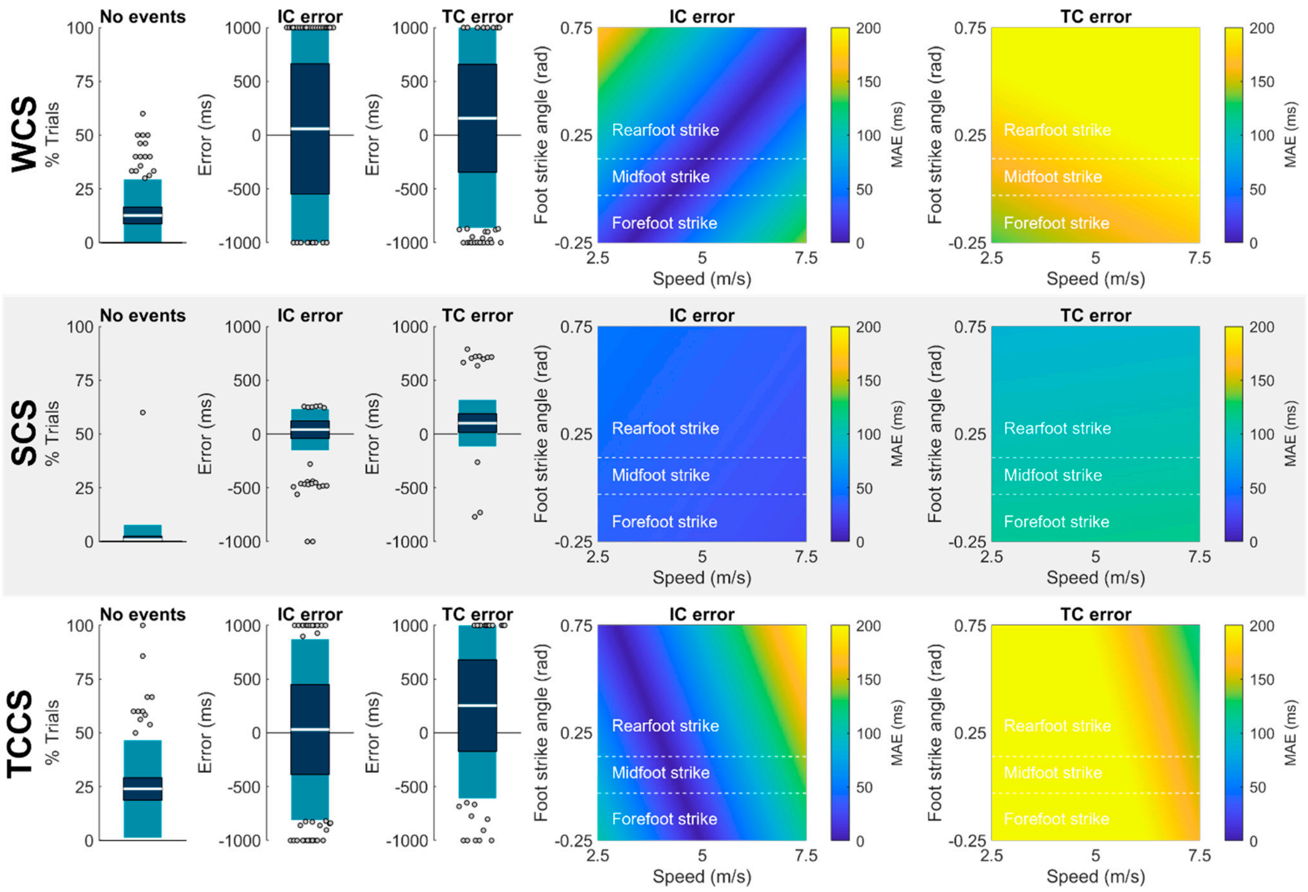

Figure S13: See Figure S2 for description.

Sinclair method

Sinclair et al. [21] placed a tri-accelerometer on the anteromedial tibia and used $a_{WCS,Y}$ to identify IC and TC gait events. Data were low-pass filtered then maxima in the ~longitudinal acceleration were identified. IC was defined as the zero-crossing point preceding each maxima while TC was defined as a plateau following the largest local maximum between longitudinal maxima. Results are shown in Fig. S15.

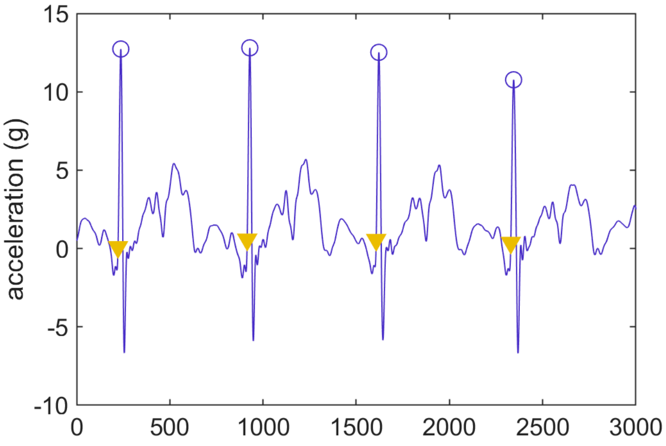

Figure S14: (A) Y-axis acceleration (proximal-distal in the SCS; ~longitudinal in the WCS; blue line) is filtered with a 4th-order 60-Hz low-pass Butterworth. Maxima separated by at least ~422 ms are identified (blue circles). Walking back from each maxima, the first zero-crossing point followed by 20 ms of positive data is defined as the IC.

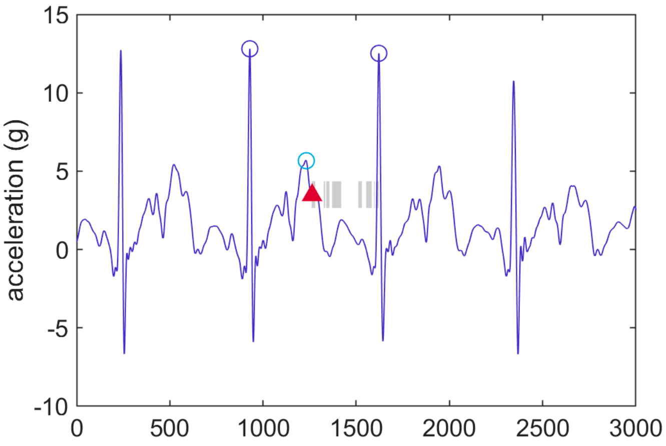

(B) To identify TC, a local maximum (light blue circle) is found between pairs of maxima. Data between this local maximum and the proceeding y-axis maximum is differentiated and portions with where the magnitude is decreasing by less than 2% of the previous frame's magnitude per second are selected (gray shading). TC is defined as the first frame of the selection occurring earliest in time with a duration of at least ~5 ms.

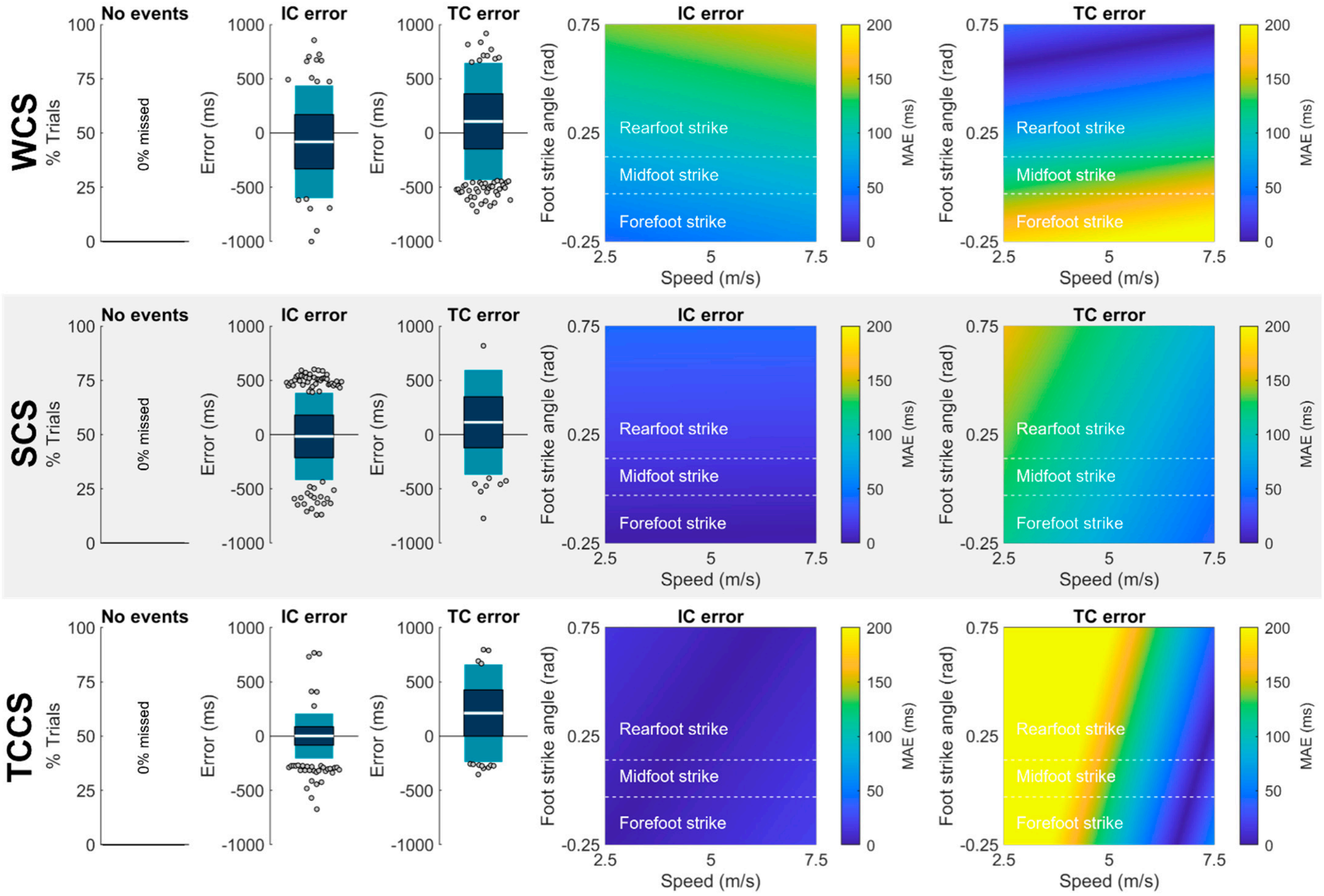

Figure S15. See Figure S2 for description.

Whelan method

Whelan et al. [22] used $a_{WCS,X}$ from a triaxial accelerometer placed on the tibialis anterior (anteromedial on the midshaft of the tibia) to identify IC events in sprinters running at up to 50% of their maximum effort. Data were first filtered using a 10 Hz low-pass Butterworth filter (order not reported; we assumed 4th). Then they reported using "peak acceleration" to define IC. Their figures, however, suggest that they defined IC using a local maximum immediately preceding a larger maximum. We developed automated code based on their figure. Results are shown in Fig. S17.

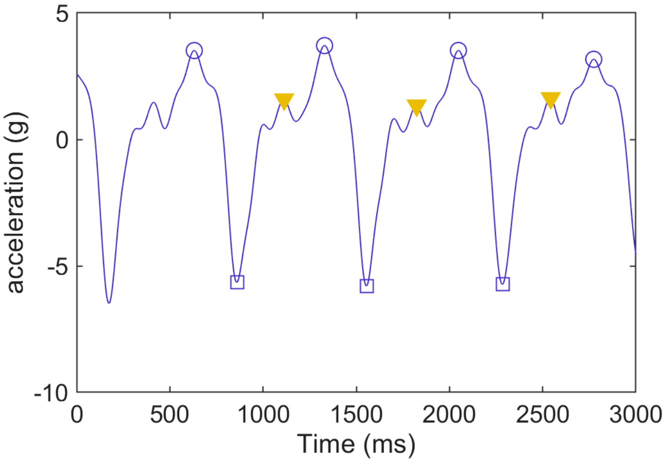

Figure S16: *X*-axis acceleration (anterior-posterior in the LCS; ~direction of progression in the WCS) was filtered using a 4th order 10 Hz low-pass Butterworth filter (dark blue line). Maxima were then found in the signal (we added a constraint that they must be separated by at least ~422 ms) (dark blue circles). The minimum value between successive maxima was then found (dark blue squares). Finally, a local maximum between the minimum and it's proceeding maximum was found and labelled as the IC (yellow triangles). TC was not identified using this method.

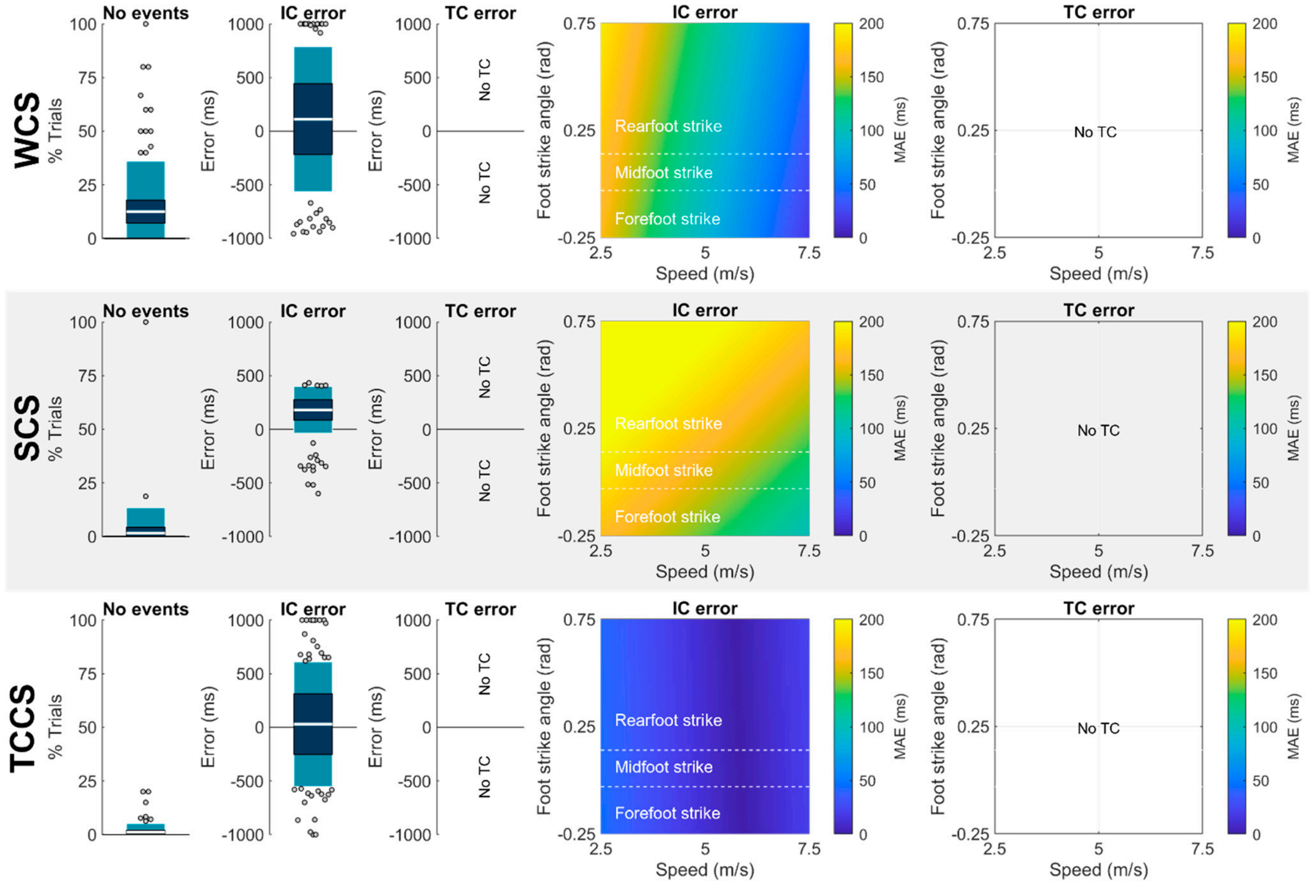

Figure S17. See Figure S2 for description.

Norris method.

Norris et al. [23] proposed a running gait event identification method where $a_{WCS,z}$ from a tri-axial accelerometer placed on the anteromedial distal tibia were filtered with a 2 Hz 2nd order low-pass Butterworth filter and a zero-crossing was used to identify IC. Results are shown in Fig. S19.

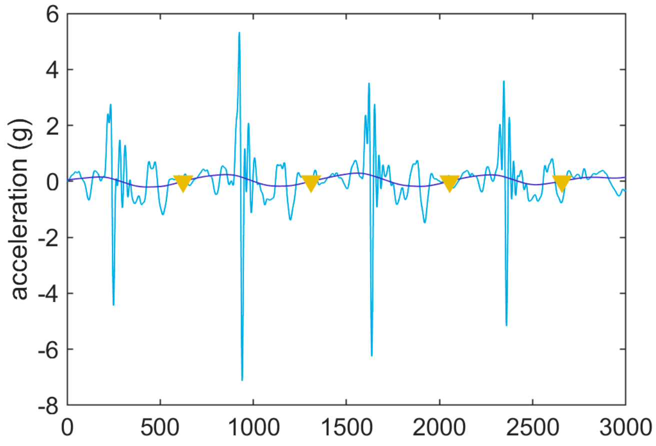

Figure S18: *Z*-axis acceleration (medial-lateral in the SCS; ~right in the WCS; light blue line) is filtered with a 2nd-order 2 Hz Butterworth filter (dark blue line). IC is defined as any positive zero-crossing point. No TC definition is provided.

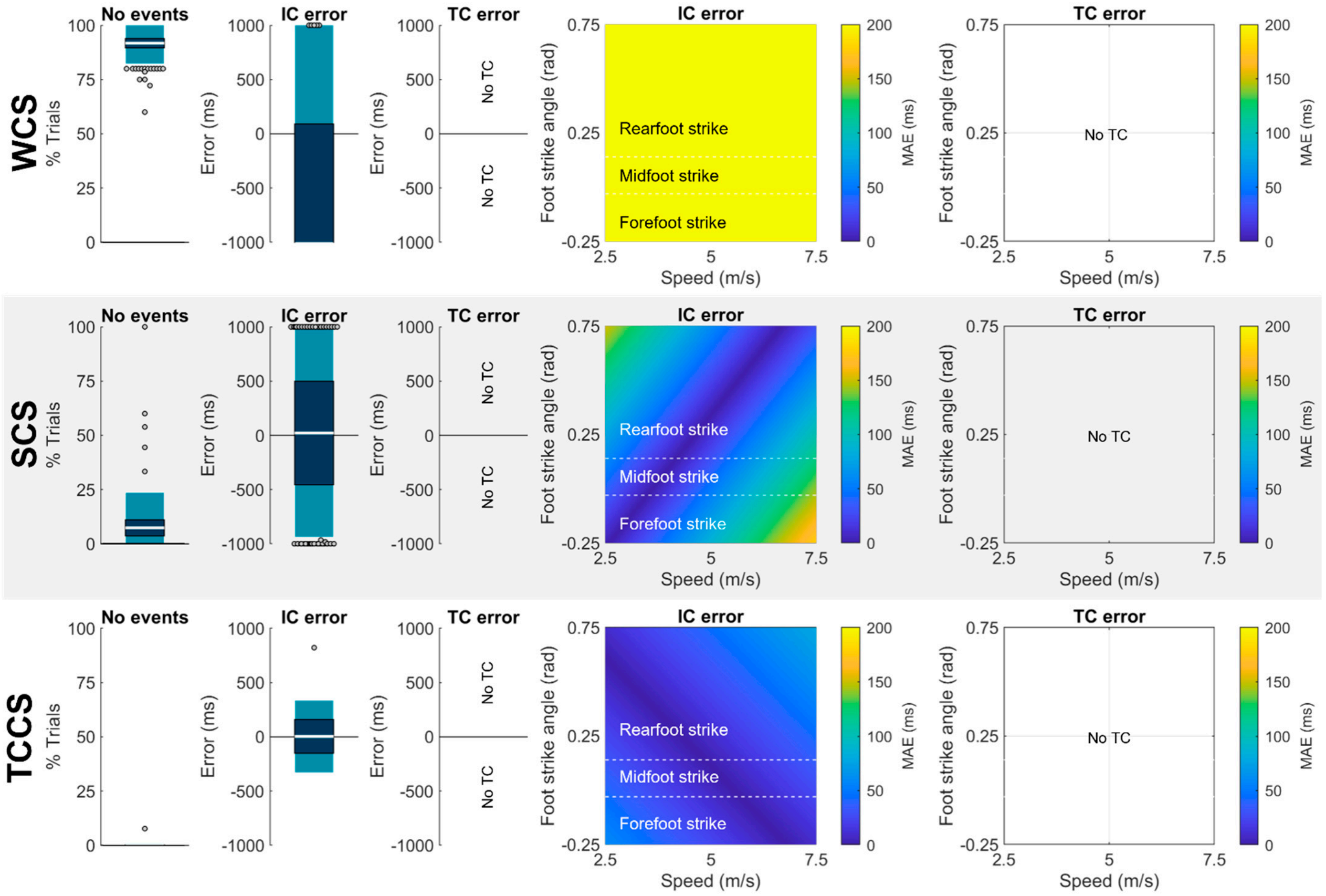

Figure S19: See Figure S2 for description.

Schmidt method.

Schmidt et al. [24] developed a gait event identification method for sprinting. It should be noted that this method was not developed with, or intended for, use across a broad range of running speeds. To identify IC and TC events, Schmidt et al. placed a triaxial IMU on the distal lateral tibia and identified points in $a_{WCS,Y}$ and $\omega_{WCS,Z}$ that met

user-defined thresholds. Given our focus on unsupervised methods, however, we adapted this code to use default thresholds. We report only the unsupervised results here, it is likely that results would improve with supervision. Results are shown in Fig. S21.

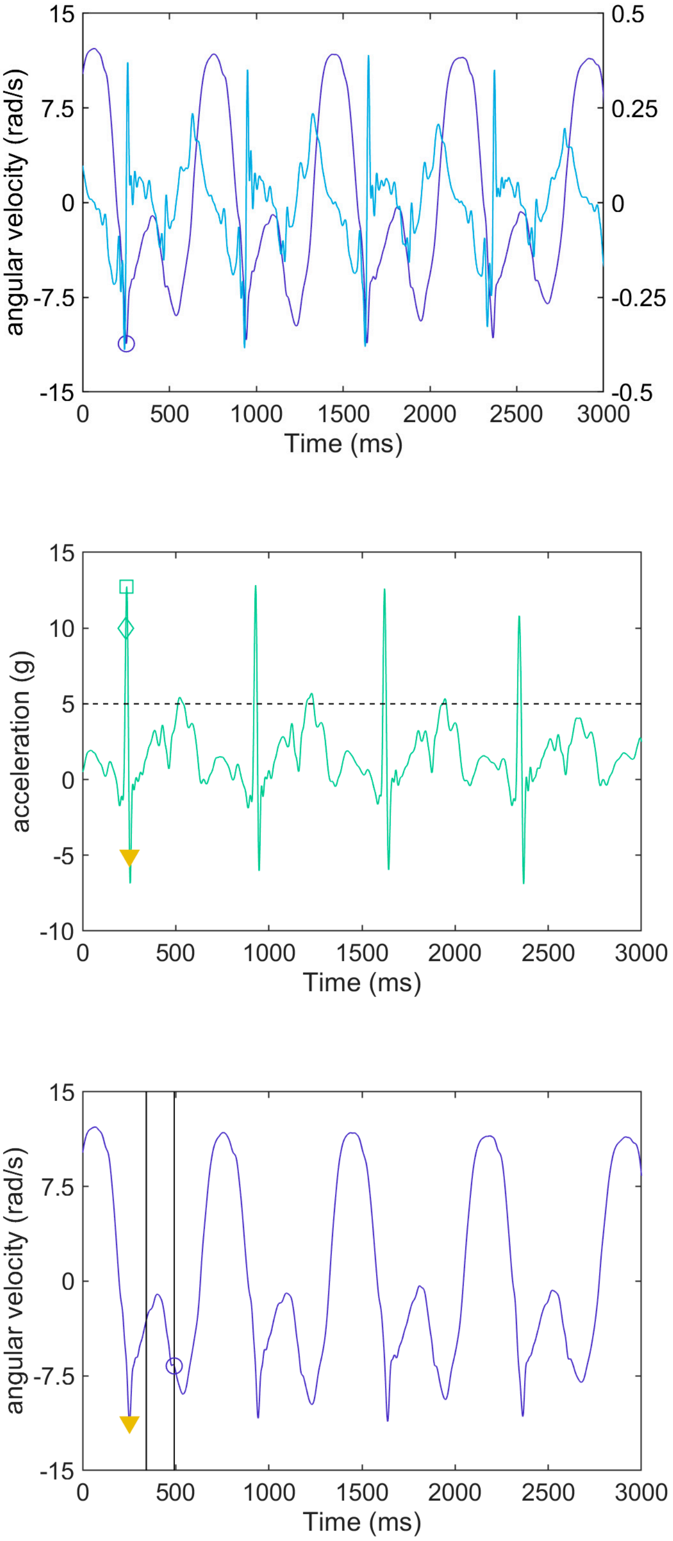

Figure S20: (A) Find the global minimum in angular velocity about the *z*-axis (medial-lateral in SCS; ~right in WCS; dark blue line). Then, walk through each frame of data and find the local minimum in angular velocity across the next 100 ms and the differential in angular velocity (angular acceleration; light blue line) for the next 50 ms. If the local minimum is equal to the global minimum or all angular accelerations are negative and the linear acceleration in the *y*-axis (proximal-distal in the SCS; ~longitudinal in the WCS; green line; **B**) exceeds a critical threshold (5 g or user-defined; dashed black line) then flag the current frame as a point of interest (In the example data, a single frame meets these criteria—the global minimum in angular velocity about the *y*-axis; dark blue circle).

(B) For each point of interest, find the maximum in *y*-axis acceleration occurring between 20 ms before to 100 ms after the point of interest (green square). Then, find the *y*-axis acceleration minimum occurring between 20 ms before the point of interest to the *y*-axis acceleration maximum (green diamond). If the minimum occurs after the point of interest label it as the IC; otherwise, label the point of interest as the IC (In this case, the point of interest was labeled as the IC; dark blue triangle).

(C) Next, look through all ICs. Within a 150 ms (or user defined) time window starting 90 ms (or user defined) after the IC (vertical black lines) find the minimum angular velocity (dark blue circle).

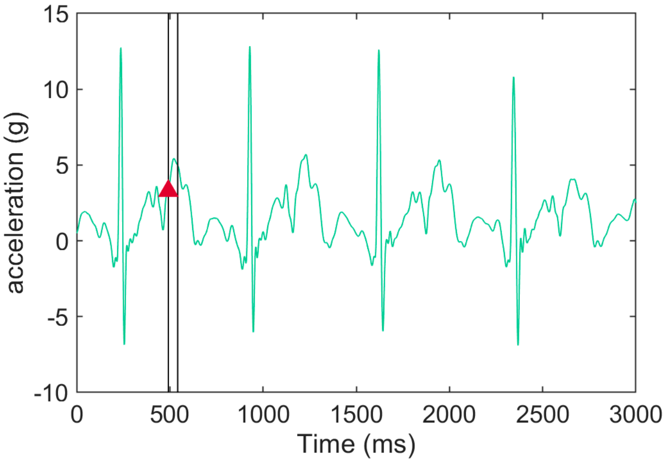

(D) Starting from this minimum, within a ~50 ms window (black vertical lines) and find the minimum y-axis acceleration (in this case, the same frame as the minimum angular velocity from the previous step). Label this as the TC (red triangle). If multiple estimated timings are obtained for a single gait event take the mean value.

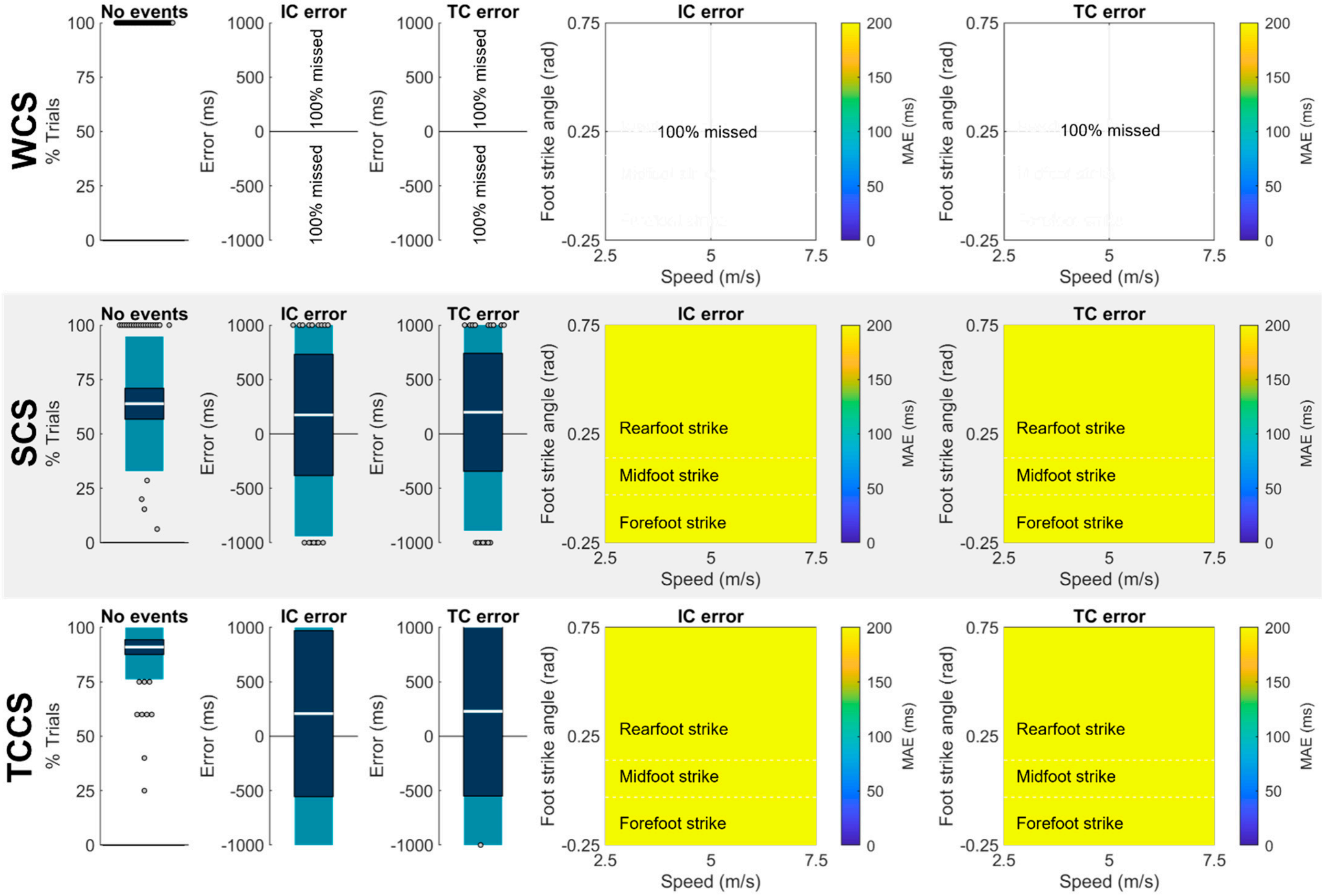

Figure S21. See Figure S2 for description.

Aubol method.

Aubol et al. [25] used $a_{WCS,resultant}$ from a triaxial accelerometer mounted on the anteromedial distal tibia to estimate IC. First, each axis' acceleration was filtered with a 70-Hz 4th-order low-pass Butterworth filter. Then features of resultant acceleration and jerk were used to identify IC. Results are shown in Fig. S23.

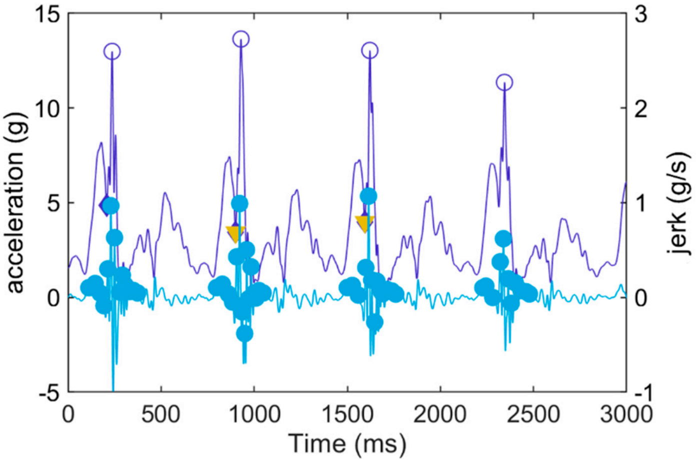

Figure S22: Tri-axial accelerations were filtered using a 70-Hz 4th order low-pass Butterworth filter and used to calculate resultant acceleration (dark blue line) and jerk (light blue line). Maxima in the resultant acceleration separated by at least ~422 ms were identified (dark blue circles; the timing constraint was a novel constraint we introduced). Next, maxima in the resultant jerk were identified, however, only maxima within ~150 ms of a resultant acceleration maximum were retained (light blue circles). Acceleration minima with a prominence of at least 0.2*the magnitude of the third largest resultant acceleration peak were found (dark blue diamonds). Any minimum with a resultant acceleration peak occurring in the preceding ~25 ms is eliminated. Looking between pairs of subsequent resultant acceleration maxima (dark blue circles), the earliest occurring minimum is labeled as the IC.

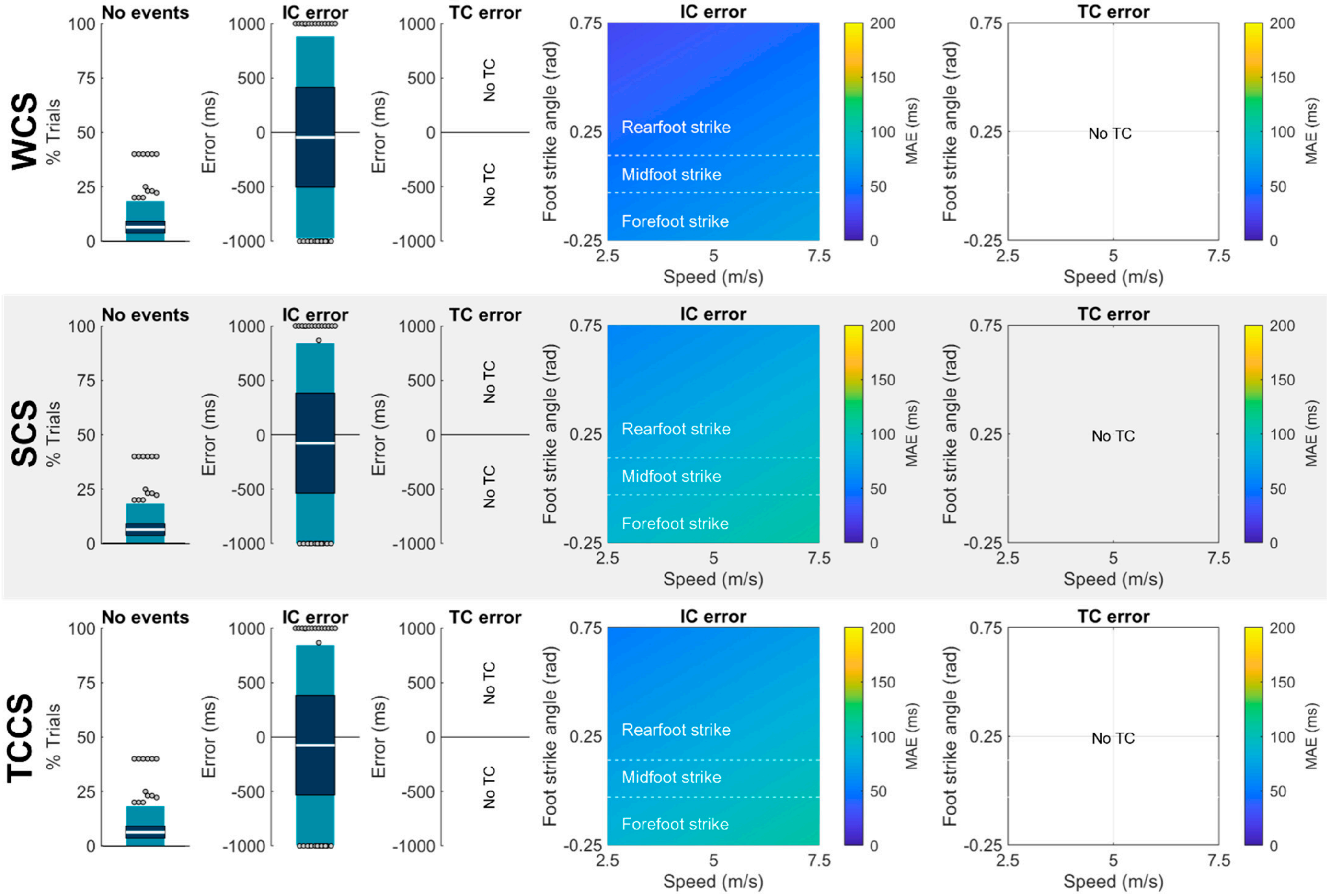

Figure S23. See Figure S2 for description.

Fadillioglu method.

Fadillioglu et al. [26] aimed to create a method capable of identifying gait events during both walking and running. To implement this method, Fadillioglu et al. placed a uniaxial gyroscope on the lateral distal tibia to capture $\omega_{WCS,z}$. The signal was low-pass

filtered then midswing peaks were identified. A complementary signal was created by subtracting the filtered signal from the original signal and then features of this complementary signal were used to identify IC and TC. Results are shown in Fig. S25.

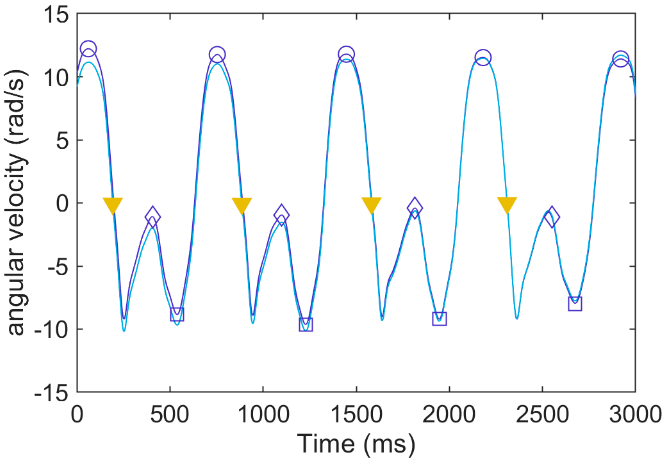

Figure S24: (A) Angular velocity about the *x*-axis (medial-lateral in the SCS and ~right in the WCS; dark blue line) is filtered with a 15 Hz 4th order low-pass Butterworth filter. Maxima with a magnitude of at least 4.63 rad/s and with a minimum separation of at least ~333 ms are identified as midswing peaks (dark blue circles). The signal is then detrended (light blue line) and for each midswing peak, the first zero-crossing following the peak is found and labelled as the IC (yellow triangles). Next, for each sequential pair of midswing peaks, the minimum value in the non-detrended signal (dark blue square) is found within a window from halfway between the peaks (dark blue diamond) to the second peak + 0.1*the time between the peaks.

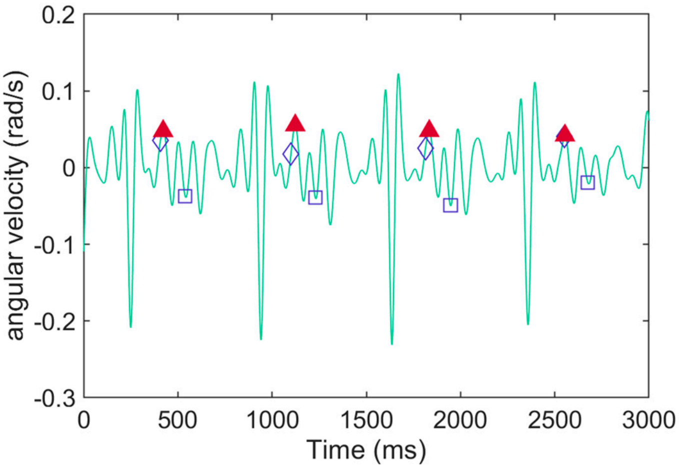

(B) Next, a complementary signal is created by subtracting the non-detrended filtered angular velocity from the unfiltered angular velocity. The complementary signal is low-pass filtered with a 2nd order 10-Hz Butterworth filter. A window of interest is defined based on the time between sequential midswing maxima: For times less than ~1000 ms (as in the example where the time is ~690 ms), TC (red triangle) is defined as the maximum complementary signal within a search window that starts halfway between midswing maxima (dark blue diamond) and ends at the negative peak (dark blue square). For times greater than or equal to ~1000 ms, TC is defined as the minimum complementary signal within a window starting halfway between midswing peaks and ending at the negative peak + 0.1*the time between the peaks.

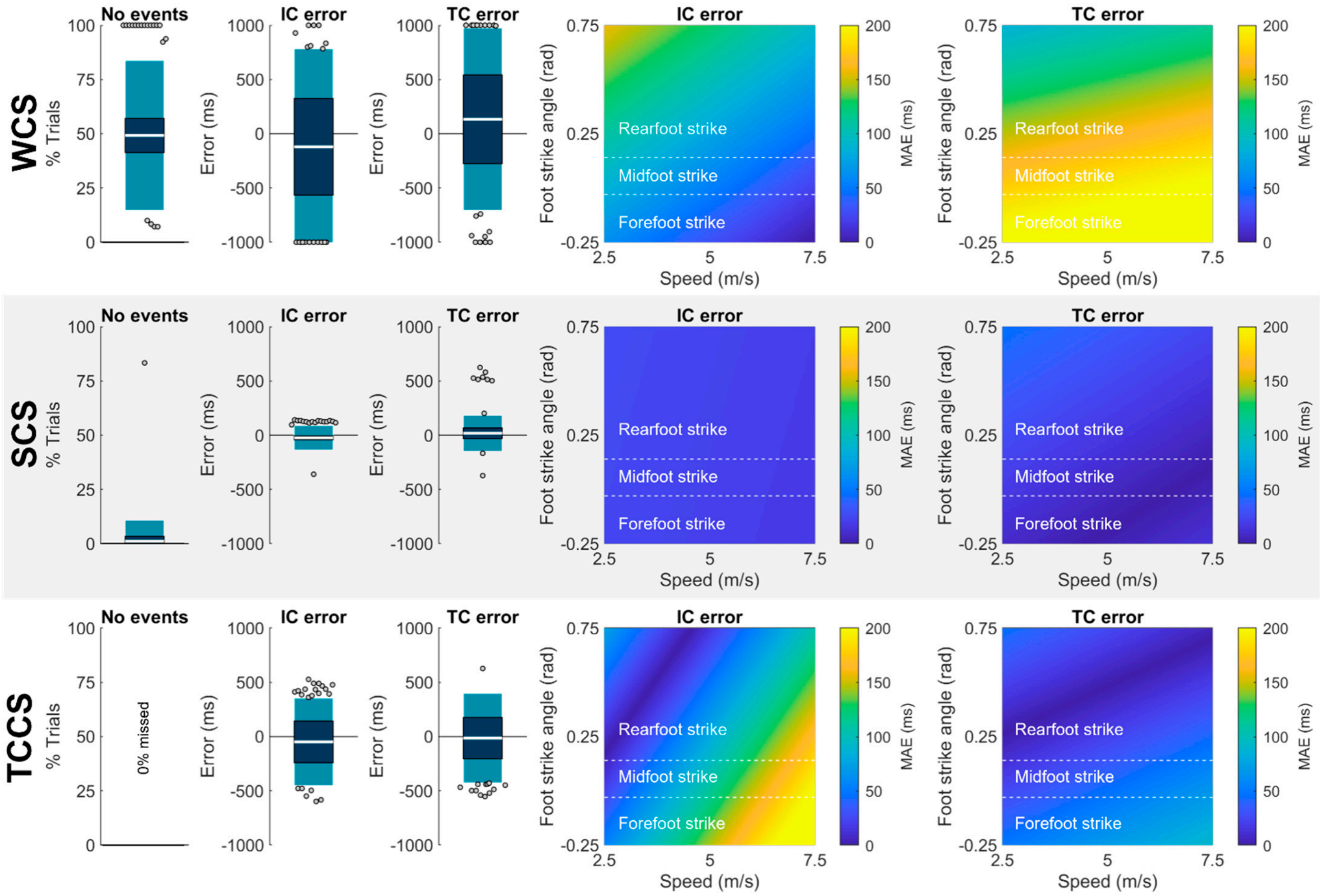

Figure S25. See Figure S2 for description.

Bach method.

Bach et al. [27] placed a tri-axial accelerometer on the anteromedial proximal tibia. They entered acceleration data into a principal component model and retained the principal component explaining the most variance for further analysis. The PC was normalized to its standard deviation and filtered and integrated to obtain velocity and position. Each signal was then entered into a machine learning algorithm and trained to estimate ground reaction forces. Estimated ground reaction forces were then used to identify IC and TC events. Results are shown in Fig. S27.

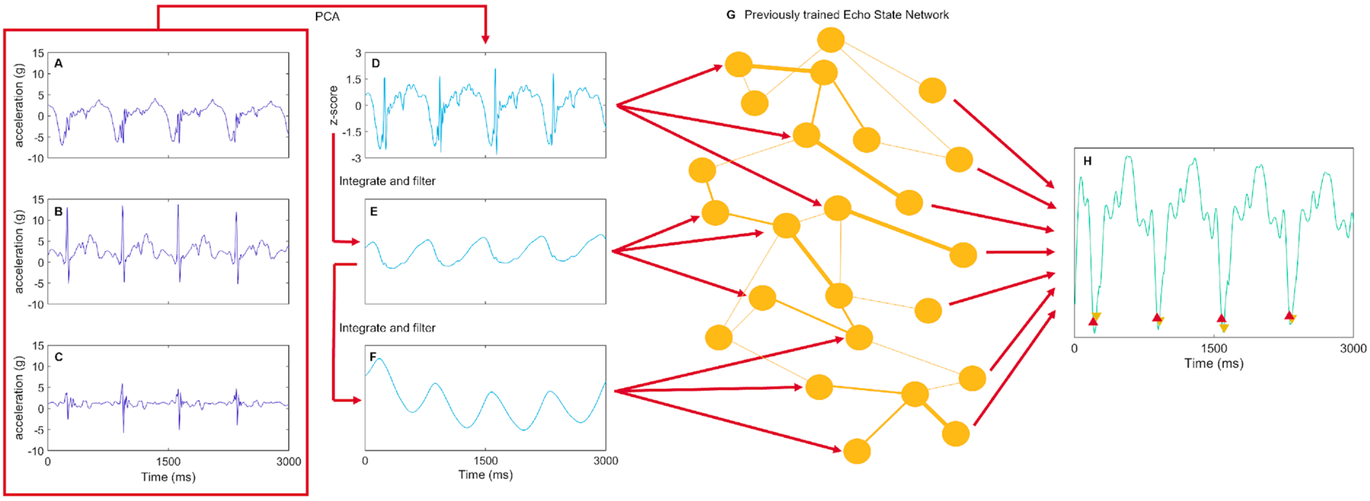

Figure S26: First, tri-axial acceleration data are resampled to match Bach et al.'s original sampling frequency **(A-C)** (the Echo State Network was trained on data collected at ~142.9 Hz and data collected at higher frequencies may have signal features that are novel to the network and degrade performance). Next, accelerations are entered into a Principal Component Analysis and the first component (explaining the most variance in the data) is retained **(D)**. The principal component "acceleration" is then normalized to its standard deviation and integrated to find principal component "velocity." The principal component velocity is high-pass filtered with a 2nd order 1-Hz Butterworth filter **(E)** then integrated to obtain principal component "position." The principal component position is then filtered using the same parameters **(F)**. Principal component acceleration, velocity, and position signals are entered into an Echo State Network that has been previously trained using Bach et al.'s published data **(G)** yielding estimated ground reaction forces **(H)** that are used to define IC and TC events (yellow and red triangles). (In this example, it appears the Echo State Network has almost captured the shape and location of four distinct stances in the ground reaction force signal, however, these distinct stances are separated by noise and large troughs that interfere with the method's ability to accurately identify IC and TC events).

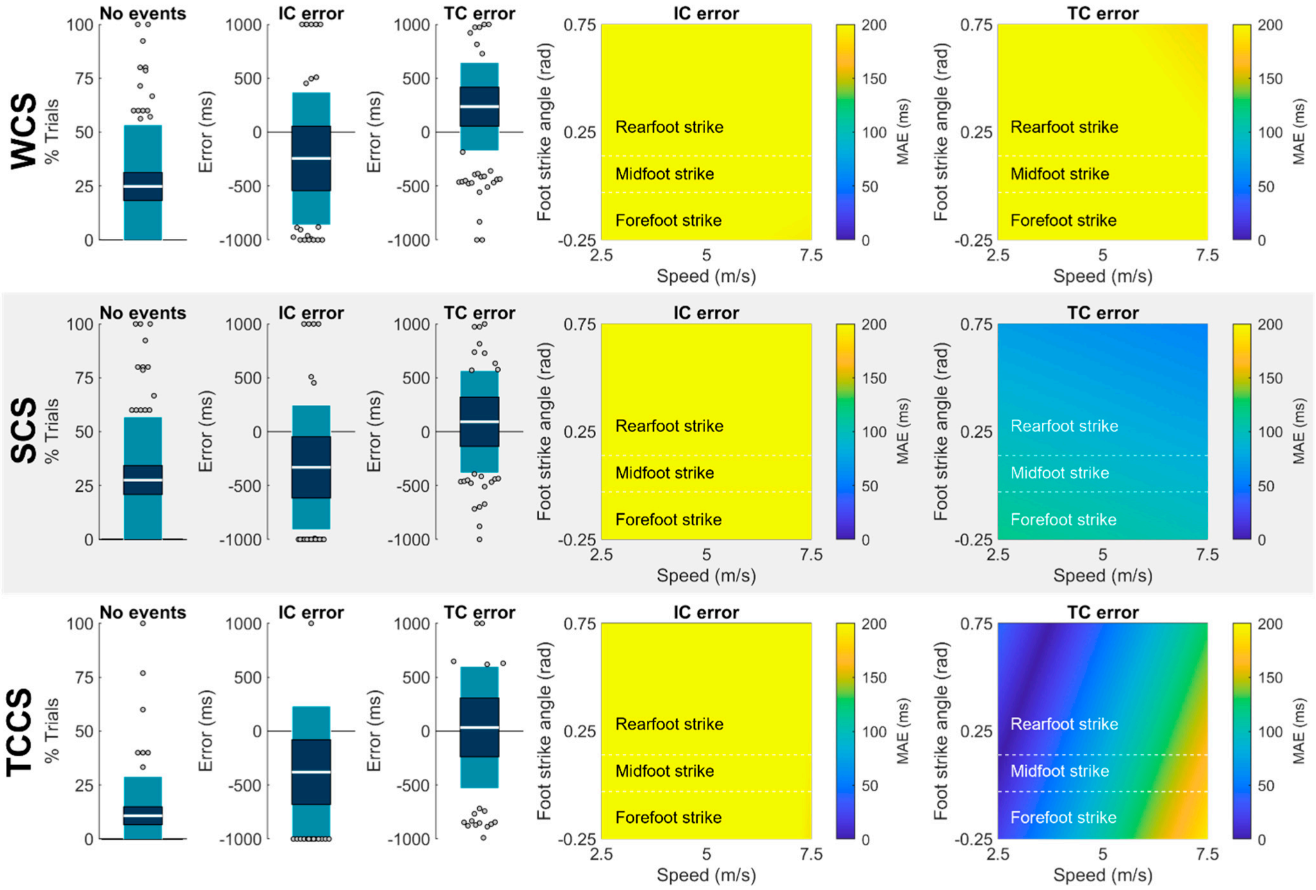

Figure S27. See Figure S2 for description.

Bach modified method

We also provide a modified version of the Bach et al. method [27]. This version addresses the large amount of negative noise we observed between the vGRF waveforms by zeroing out any vGRF values below zero then multiplying the vGRF by negative one before feeding it into Bach et al.'s gait event estimation algorithm (to cancel out an unexplained negative one multiplication that method performs). Results are shown in Fig. S28.

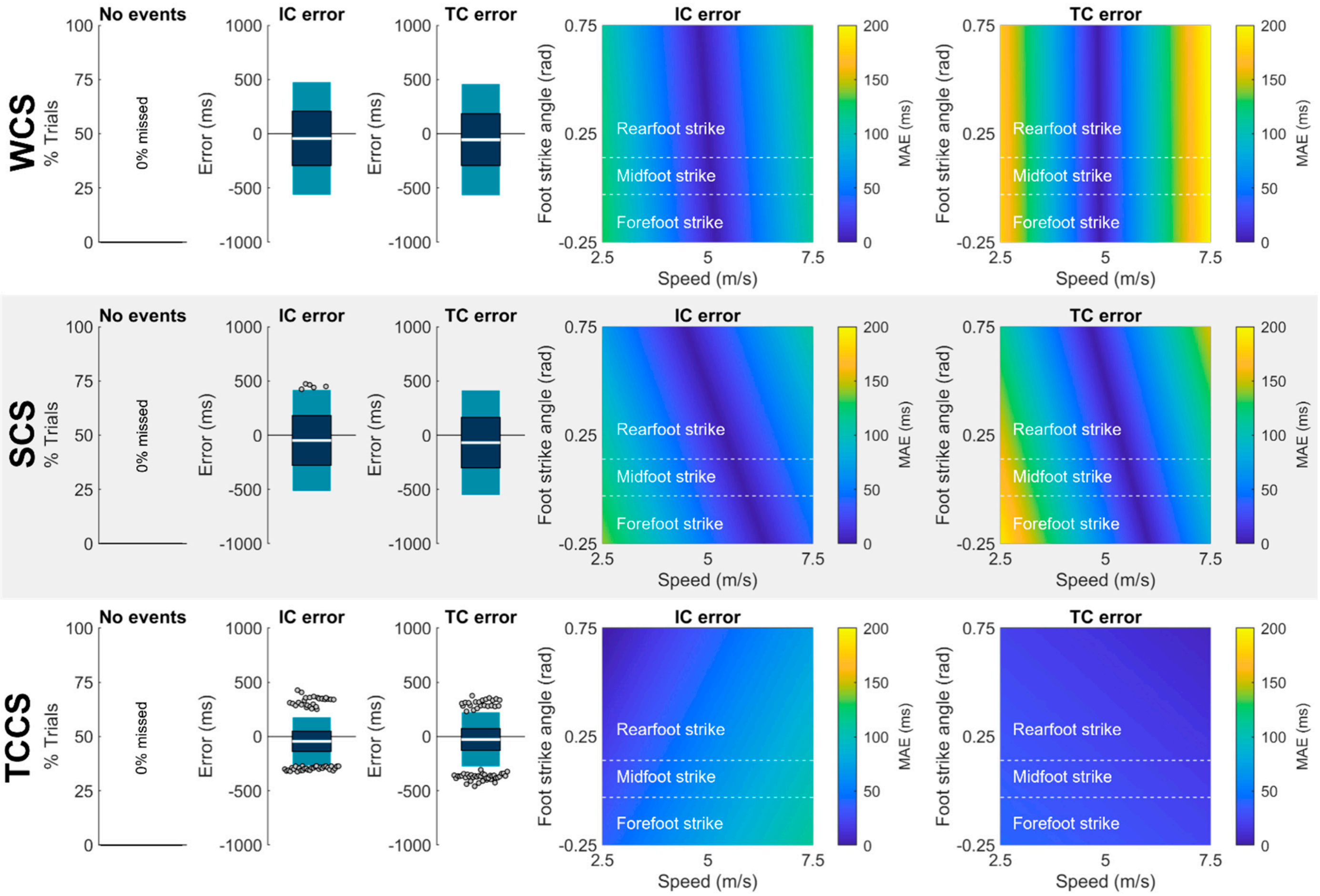

Figure S28. See Figure S2 for description.

Lower back-mounted wearable methods

Auvinet method.

Auvinet et al. [28] identified specific features associated with gait events in the signal of a tri-axial accelerometer placed on the mid-sagittal lumbar spine. Based on their simultaneous recording of acceleration and video data they identified IC as the start of a large peak in $a_{WCS,Y}$ co-occurring with the start of a deep minima in the $a_{WCS,X}$. TC was identified as the end of the peak in $a_{WCS,Y}$. Right and left side were also identifiable based on $a_{WCS,Z}$. We developed a novel automated code to identify gait events based on these features. Results are shown in Fig. S30.

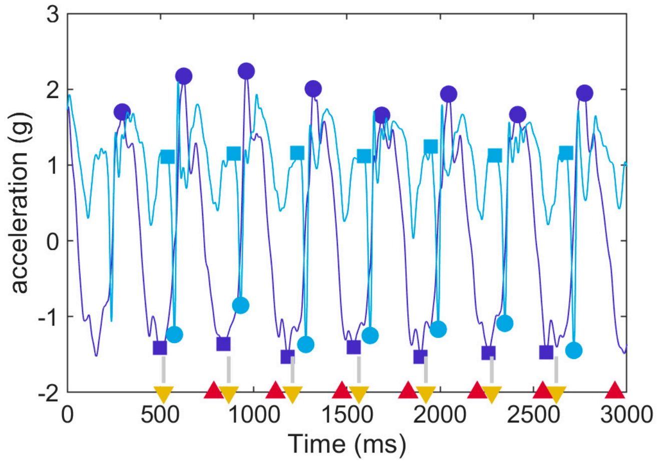

Figure S29: Identify y-axis (proximal-distal in the SCS, ~longitudinal in the WCS; dark blue line) maxima separated by at least ~422 ms (dark blue circles). Then look for the minimum value between successive maxima (dark blue squares). Next, in the x-axis acceleration (anterior-posterior in the SCS, ~direction of progression in the WCS; light blue line) find minima (light blue circles) occurring between the y-axis minimum and its proceeding maximum. Then, walk back until values start decreasing (light blue squares). Define IC (yellow triangles) as the average frame between the points where y-axis minimum and where x-axis acceleration started decreasing. To identify TC (red triangles), look for the first frame following each y-axis maximum where y-axis acceleration magnitude falls below -1 g. Finally, to determine side, take the mean value of the z-axis acceleration (medial-lateral in ~SCS, ~right in WCS) in a window extending ~10 ms to each side of the IC (gray bars). If the magnitude is less than 0, call it a left stance. If the magnitude is more than 0, call it a right stance.

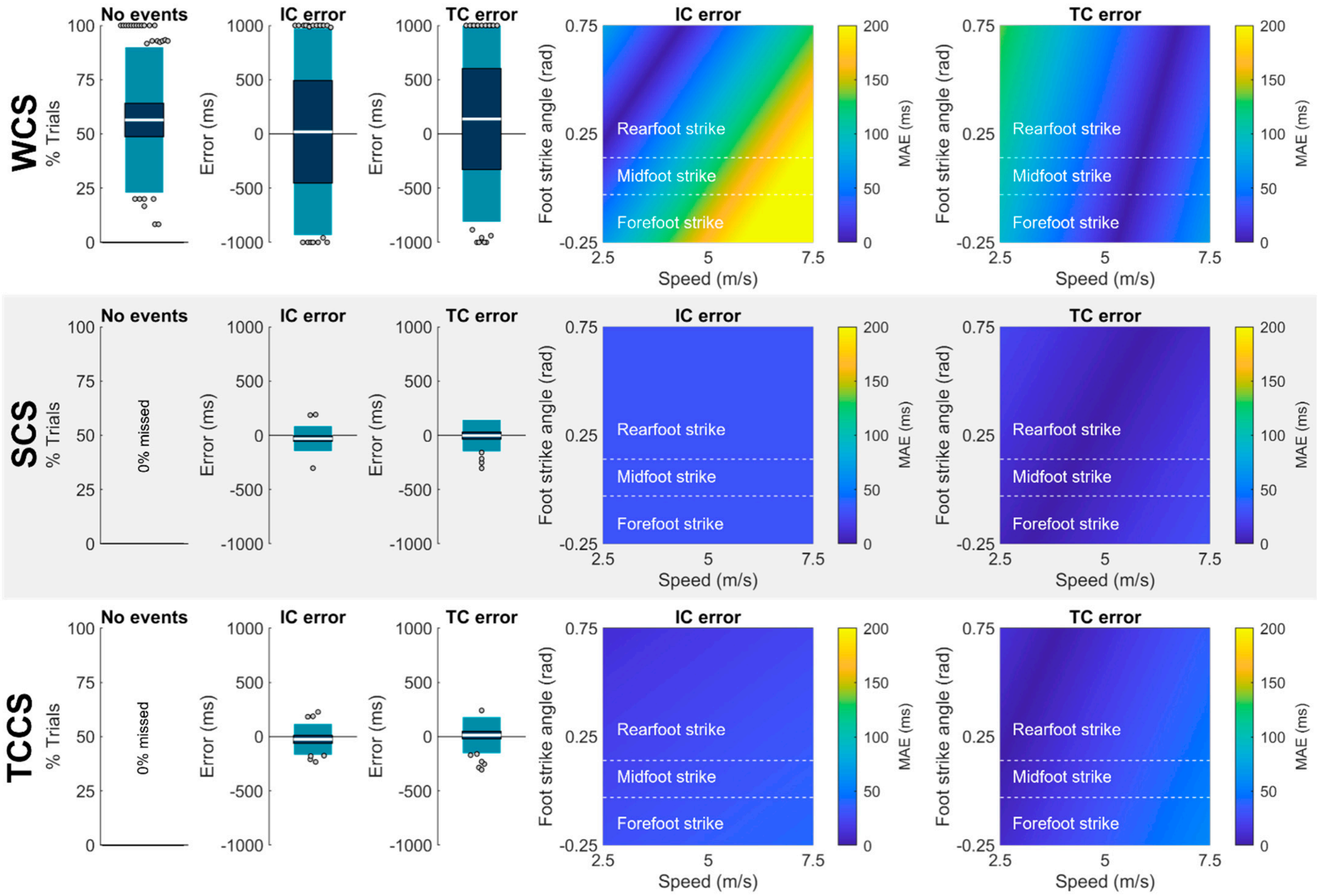

Figure S30. See Figure S2 for description.

Lee method.

Lee et al. [29] placed an IMU with tri-axial accelerometer on the sacrum. They identified IC and TC as maxima in $a_{WCS,x}$ and used $a_{WCS,z}$ to identify side. Results are shown in Fig. S32.

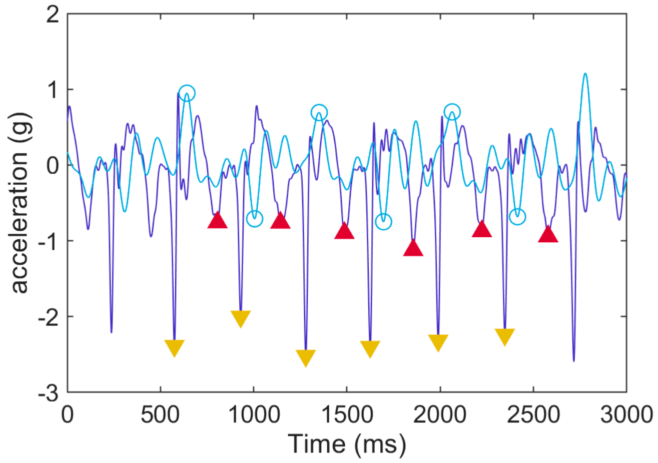

Figure S31: To identify IC, find minima in the x-axis acceleration (anterior-posterior in SCS, ~direction of progression in WCS) separated by at least ~211 ms. Label this point the IC (gold triangles). Look from one IC plus ~85 ms to the proceeding IC and find a secondary x-axis minima. Label this point the TC (red triangles). To identify stance side, low-pass filter z-axis accelerations (medial-lateral in SCS, ~right in WCS) with a 10-Hz 4th order Butterworth filter. Then, looking between successive IC and TC events, find positive and negative peaks. If the absolute value of the negative peak is greater label the stance as left. If the positive peak is greater label the stance as a right.

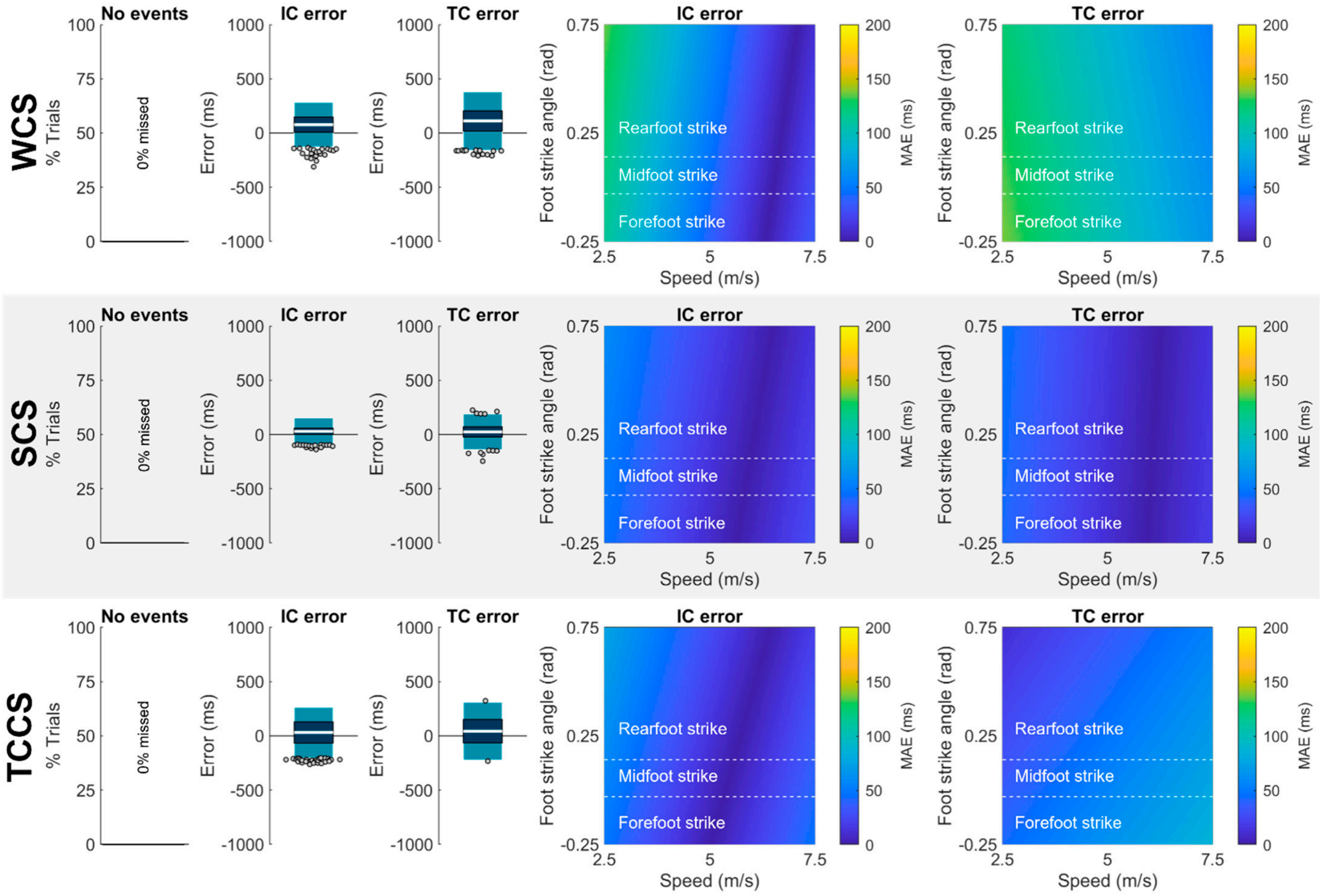

Figure S32. See Figure S2 for description.

Wixted method.

To characterize acceleration signals related to gait events, Wixted et al. [30] simultaneously recorded accelerations from L3-L4 and pressures from in-shoe insoles. They observed that IC occurred around the time of a negative peak in $a_{WCS,X}$ (similar to Lee et al. [29]) while TC occurred around the time of a negative zero-crossing in $a_{WCS,Y}$ (similar to Auvinet et al. [28]). Results are shown in Fig. S34.

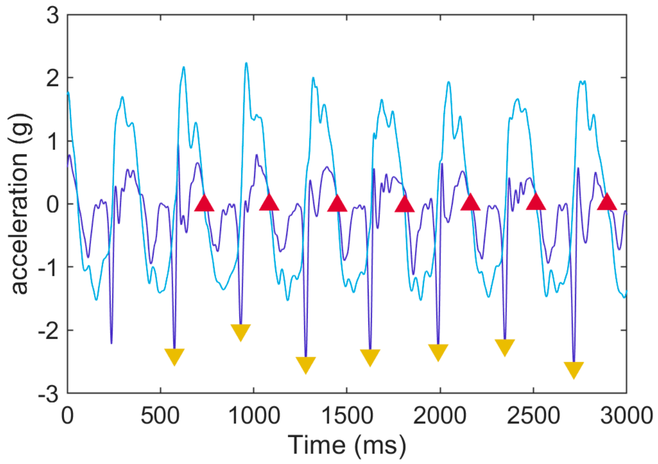

Figure S33: To identify IC, find negative peaks in the x-axis acceleration (anterior-posterior in SCS, ~direction of progression in WCS; dark blue line) separated by at least ~211 ms. Label these points the ICs (gold triangles). Starting ~ 85 ms after each IC, find the first y-axis acceleration value (proximal-distal in the SCS, ~vertical in the WCS; light blue line) that goes below 0. Label this point the TC (red triangles).

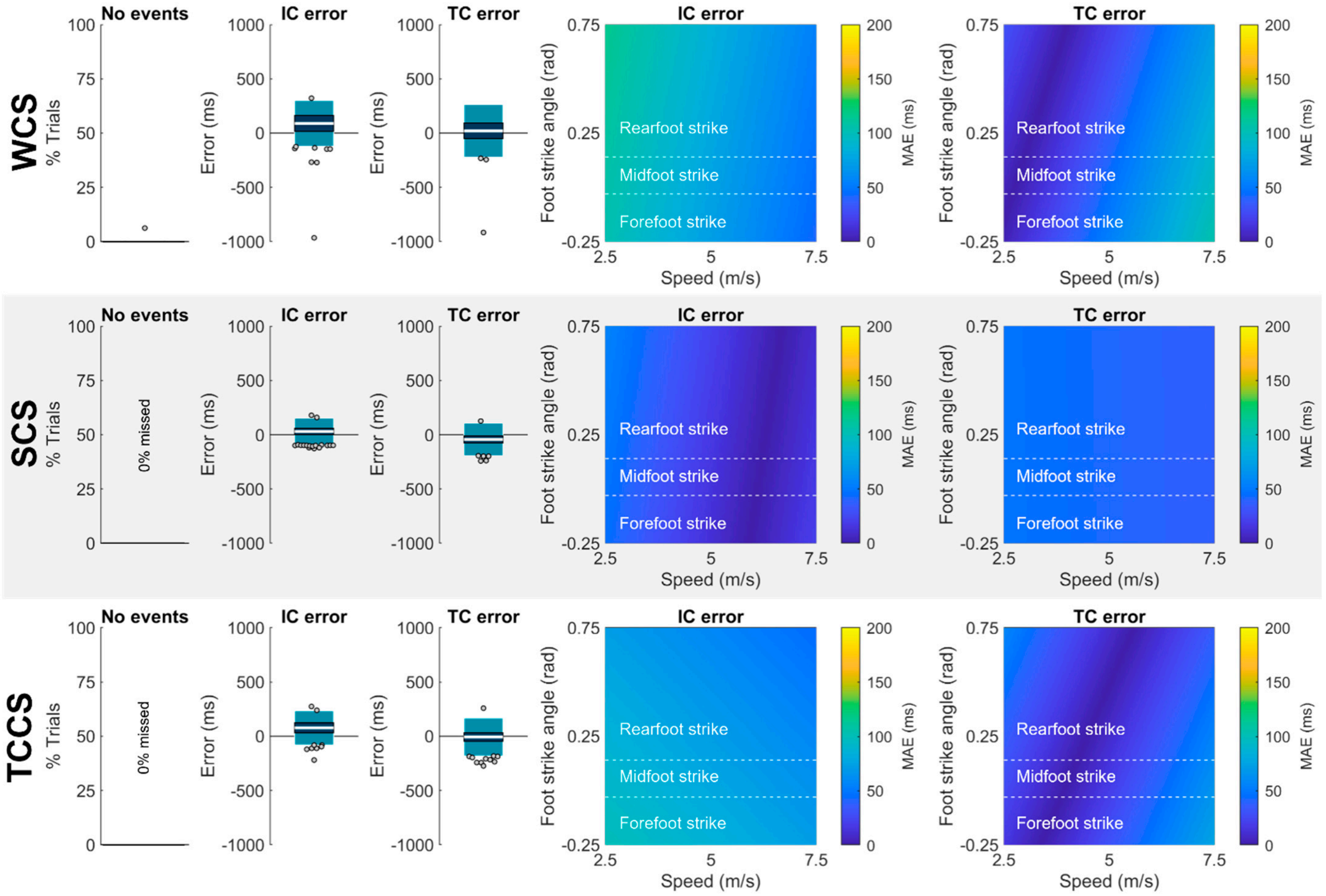

Figure S34. See Figure S2 for description.

Bergamini method.

Bergamini et al. [31] placed a tri-axial IMU on the lumbar spine and used Luo et al.'s wavelet-mediated differentiation technique [32] to double differentiate $\omega_{WCS,resultant}$. Positive peaks in the double differentiated signal are used to define IC while minima are used to define TC. Results are shown in Fig. S36.

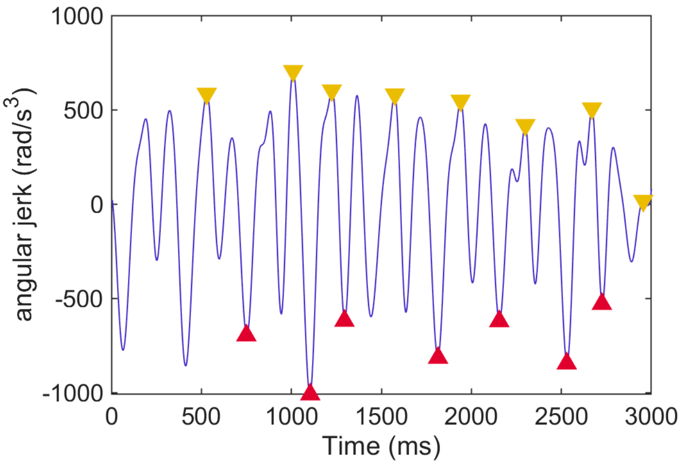

Figure S35: Angular velocity is resampled to 200 Hz, matching Bergamini et al.'s original paper. Then resultant angular velocity is calculated and double differentiated using 4-level quadratic spline discrete wavelet differentiation as described by Luo et al. [32] (dark blue line). Positive peaks separated by at least ~211 ms are found in this new angular jerk signal and are labelled as IC (yellow triangles). Minima between each successive pair of positive peaks are found and labelled as TCs (red triangles).

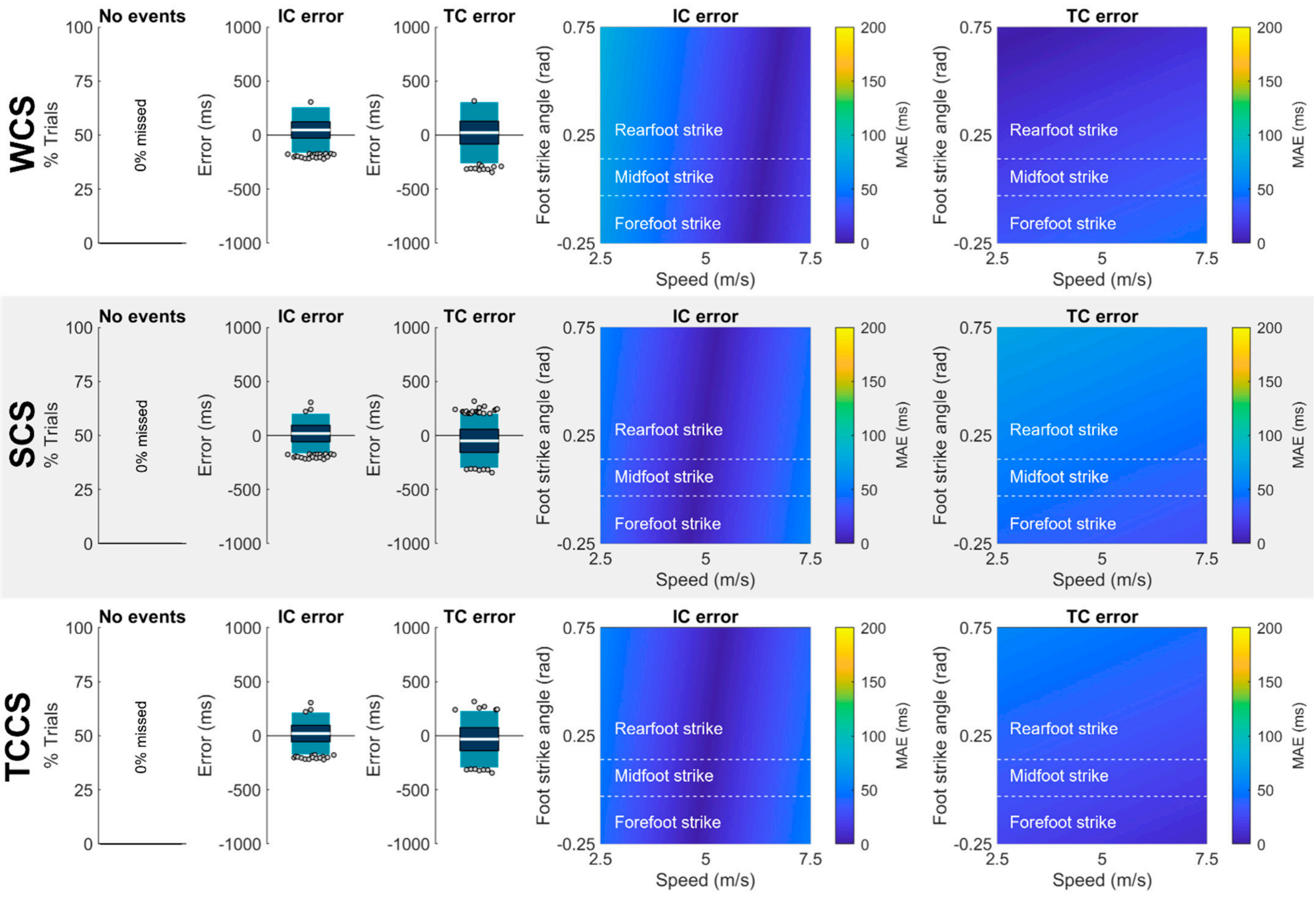

Figure S36. See Figure S2 for description.

Benson method.

Benson et al. [33] provide several well-documented functions to identify gait events from acceleration profiles of wearables mounted on the foot or sacrum. Here, we've minimally adapted their sacrum function to work with the same inputs and provide same outputs as other methods in this package. Results are shown in Fig. S38.

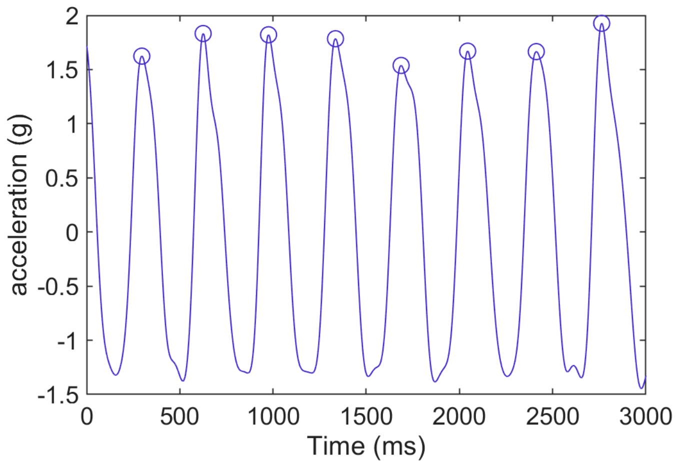

Figure S37: (A) First, accelerations are low-pass filtered with a 10-Hz 4th order Butterworth filter. Then positive peaks separated by at least ~250 ms are found in the *y*-axis acceleration (proximal-distal in the SCS, ~vertical in the WCS; dark blue line).

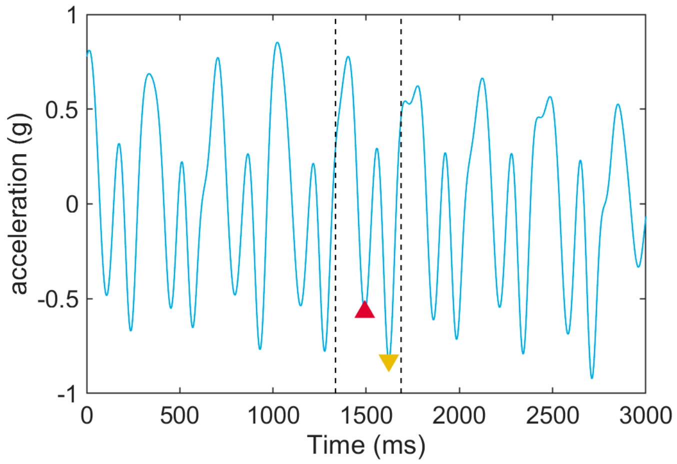

(B) Next, find negative *x*-axis (anterior-posterior in the SCS, ~direction of progression in the WCS; light blue line) acceleration peaks between each pair of successive *y*-axis peaks (vertical black lines). If there are multiple negative *x*-axis peaks, then rank them 1…n by both magnitude (largest to smallest) and by timing (latest to earliest). IC (yellow triangle) is defined as the peak with the lowest mean magnitude and timing ranking. If there is a tie in mean rankings, the later peak is accepted as IC. Then, look from the preceding *y*-axis peak to IC - ~100 ms and accept the latest occurring negative *x*-axis peak as the TC (red triangle). Note, if no negative *x*-axis peaks can be identified, find the *x*-axis minimum and label it as the IC. Then find the *x*-axis maximum between the preceding *y*-axis peak and the IC. Find the peak *x*-axis jerk between the *x*-axis maximum and the IC.

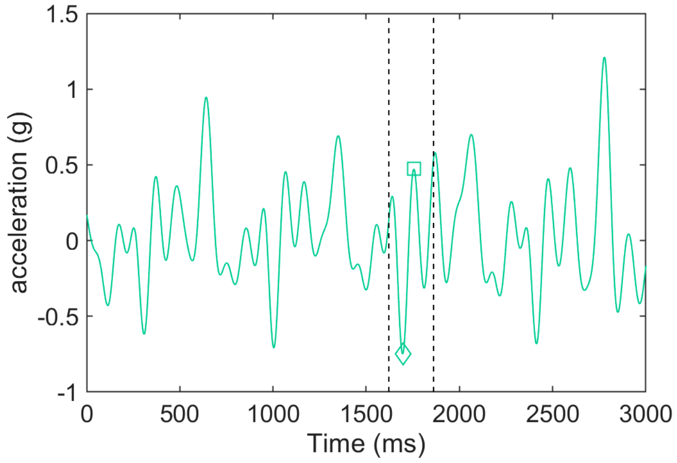

(C) To identify stance side, find the largest positive (green square) and negative (green diamond) peaks in the *z*-axis acceleration (medial-lateral in the SCS, ~right in the WCS; green line) during each stance (vertical black lines). If there is no positive (rightward) peak, then set as a left stance (and vice versa). If both positive and negative peaks exist, if the positive peak is closer to TC than to the negative peak, set as a left step; otherwise, if the negative peak occurs within ~15 ms of the IC, find a new negative peak between the current negative peak and the TC; otherwise, set as a left stance.

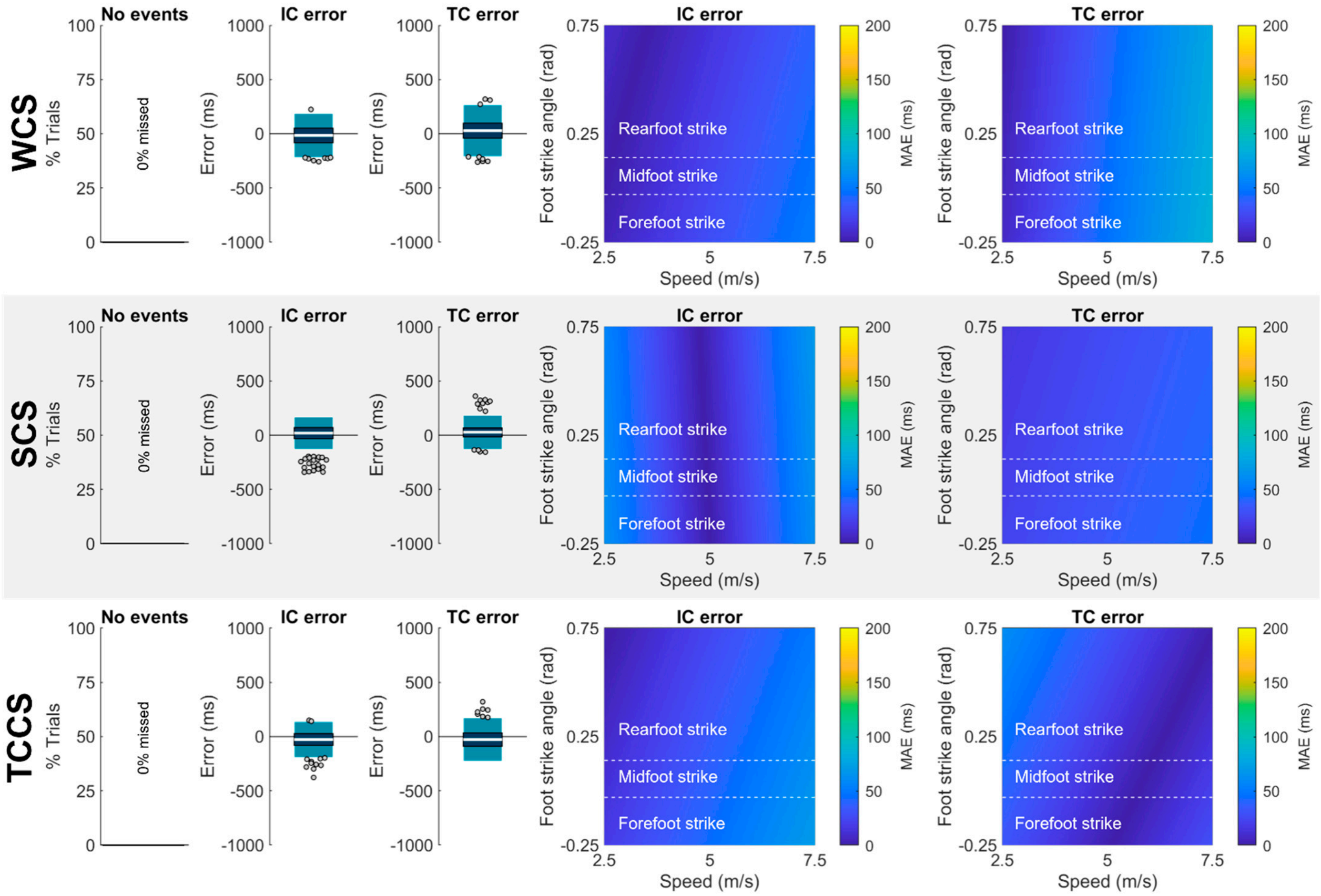

Figure S38. See Figure S2 for description.

Reenalda method.

Reenalda et al. [34] placed an IMU on runners while they ran a treadmill using different foot strike patterns. They defined IC as the peak downward velocity of the pelvis by integrating $a_{GCS,Y}$ (obtained using proprietary algorithms provided by the IMU manufacturer). Results are shown in Fig. S40.

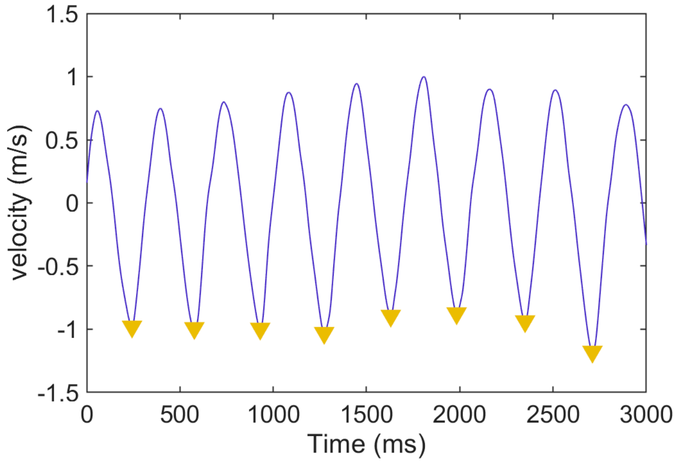

Figure S39: y-axis acceleration (vertical in the GCS, proximal-distal in the SCS, and ~vertical in the WCS) is integrated to obtain y-axis velocity. Negative peaks are found and labelled as IC. We added an additional constraint that these peaks must be separated by at least ~211 ms. No method is described to determine TC or stance side.

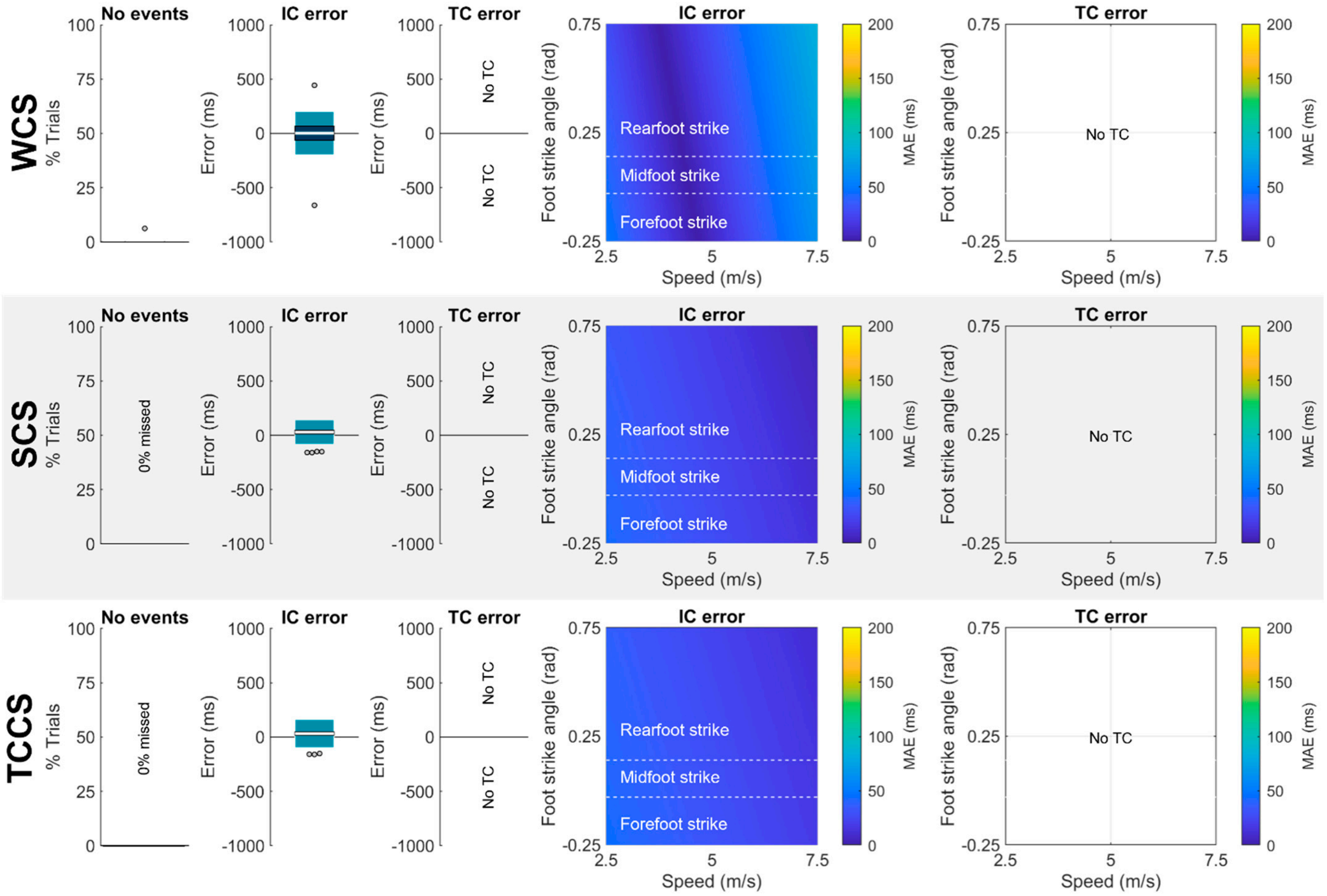

Figure S40. See Figure S2 for description.

IMU data processing

An overview of our IMU data processing is provided in the main text. Here, we expand on those processing details. After collecting raw data from each IMU, data were downloaded and processed offline using the following steps: (1) Calibration, (2) Quiet period identification, (3) Bias removal, (4) Saturation correction, (5) Low-pass filtering, (6) Drift correction, (7) Orientation estimation and gravity subtraction, and (8) Coordinate system transformation. After these processing steps each of the 642 trials we analyzed was segmented using the speed gate signals (to include data between the times when the participant crossed the gate 2.5 m before force plate center and the gate 2.5 m after force plate center) and entered into each of the 21 individual methods as an n × 7 matrix (with n rows representing the number of frames and the 7 columns representing time stamps, 3 acceleration axes, and 3 angular velocity axes). This resulted in a total of 642 trials × 21 methods = 13482 possible events for comparison to the corresponding 642 ground truth force plate events for each IC, TC, and stance side.

1. *Calibration*

All IMU data collected during this experiment were corrected with IMU-specific calibration matrices. These matrices were calculated by conducting a calibration procedure that ensured each IMU accurately expressed accelerations and angular velocities in an orthogonal coordinate system oriented square to the IMU housing.

Each IMU was secured to a centrifuge (ClearPath MCVC, Teknic, Victor, USA) with custom 3-D printed jigs (SOLIDWORKS 2019, Dassault Systèmes, Vélizy-Villacoublay, France) and calibrated in 6 orientations at 16 known accelerations (from 0 - 41.42 g where 1 g = 9.8 m/s^2 [35, 36]) and angular velocities (from 0 - 78.54 rad/s). Adapting methods from Coolbaugh et al. [37], known data (K) from the centrifuge and measured data (M) from the IMU were used to calculate 3 × 7 calibration matrices for each IMU (C; 3 signed magnitude terms, 3 absolute magnitude terms, and one bias term per axis) and quantify sensor accuracy with a hold-back procedure after subtracting out biases observed during a quiet period (B).

$$C^*(M + B) = K$$

One potential limitation of this procedure is that it treats each triaxial sensor independently (primary accelerometer, secondary accelerometer, and gyroscope) and assumes their values do not affect each other. This assumption was tested while piloting this calibration procedure by quantifying inter-sensor dependencies between the primary accelerometer and gyroscope and between the secondary accelerometer and gyroscope. Observed dependencies were negligible and independent sensor calibration matrices yielded the best results; thus, we felt confident using this approach (which avoids the indeterminacy of the primary and secondary accelerometer having the same K values).

After calibration, IMU primary accelerometer errors were ≤ 0.01±0.04 g, secondary accelerometer errors were ≤ 0.05±0.07 g, and gyroscope errors were ≤ 0.01±0.01 rad/s.

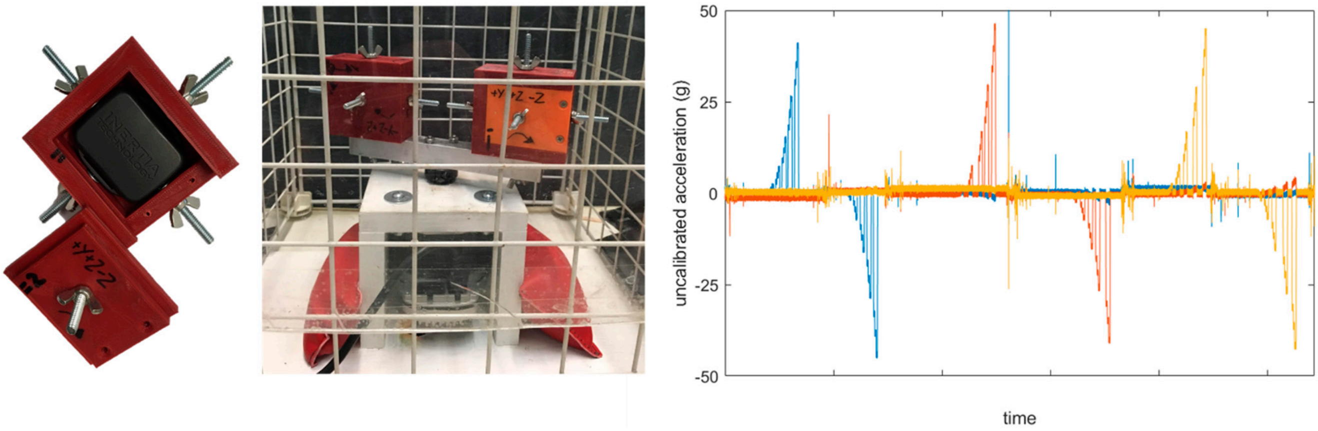

Figure S41. **Left**: : An IMU in it's 3-D printed housing. Computer-aided design software was used to ensure IMUs were friction fit square to their housing. **Middle**: Two IMUs in their 3-D printed housings mounted on the centrifuge. IMUs were checked for square with an engineer's square and level with a bullseye level. **Right**: Example of measured triaxial accelerations for the secondary accelerometer (M). The 16 accelerations being applied to the IMU in each of 6 orientations correspond to known (K) values from the centrifuge. Accelerations between each orientation correspond to the IMU being repositioned on the centrifuge and checked for square and level.

2. *Quiet period identification*

Quiet periods were identified throughout data collection (e.g., participant resting, participant preparing at the start of the runway, participant standing while receiving instruction) and used to periodically check for changes in bias (as bias can vary with battery life and temperature) and reset orientation algorithms (as orientation estimates are prone to drift over prolonged periods; discussed further below). These quiet periods were defined as any period where…

$\omega_{resultant} < 0.5\ rad/s$ and

$$j_{resultant} < 0.01\ m/s^3$$

…for at least 100 ms.

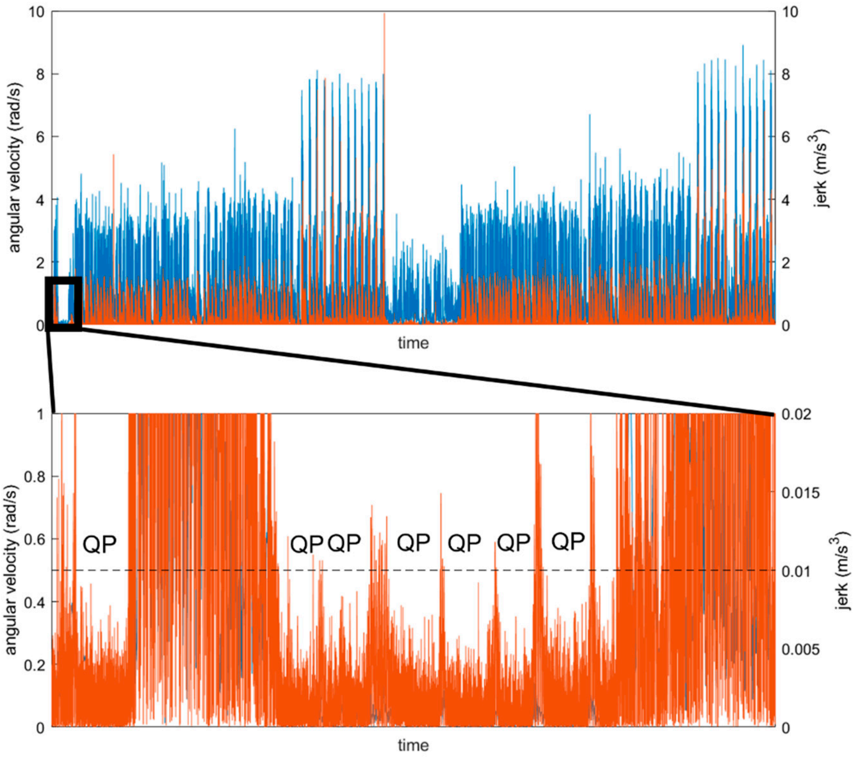

Figure S42. **Top**: Resultant angular velocity (blue) and jerk (orange) at the sacrum across an entire data collection for the randomly selected example participant. **Bottom**: Zoomed in to show quiet periods (QP) where resultant angular velocities are < 0.5 rad/s and resultant jerks are < 0.01 m/s^3 for at least 100 ms. Thresholds noted with the dashed horizontal line.

3. Bias removal

When the IMU is quiet, we know that it is not accelerating or rotating and thus, the only thing loading the axes should be the gravity vector. Based on this knowledge we can create a temporary inertial coordinate system based on gravity:

$$Y = \frac{\sum_{first\ quiet\ frame}^{last\ quiet\ frame} a_{calibrated}}{n_{quiet\ frames}}$$

We can express Y as a unit vector, then make X and Z orthogonal unit vectors (with arbitrary sense). Using these vectors, we can create a temporary rotation matrix that will align our data with gravity:

$$R_{temp} = \begin{bmatrix} X \\ Y \\ Z \end{bmatrix}$$

We can then express our data in this temporary inertial coordinate system (and given our calibration we know that the axes of the accelerometer and gyroscope are exactly aligned so the same rotation matrix can be used for both):

$$a_{temp} = R_{temp} * a_{calibrated}$$

Given the IMU is quiet, in every frame a_{temp} and ω_{temp} should now equal [0 1 0] g and [0 0 0] rad/s, respectively. Thus, we can calculate bias (B) in acceleration and angular velocity as the average deviation from those values across the quiet period:

$$B_a = \frac{\sum_{first\ quiet\ frame}^{last\ quiet\ frame}(a_{temp} - [0\ 1\ 0])}{n_{quiet\ frames}}$$

We can then remove bias and re-express our data in its original coordinates:

$$a_{debias} = a_{calibrated} - R_{temp}^{-1} B_a$$

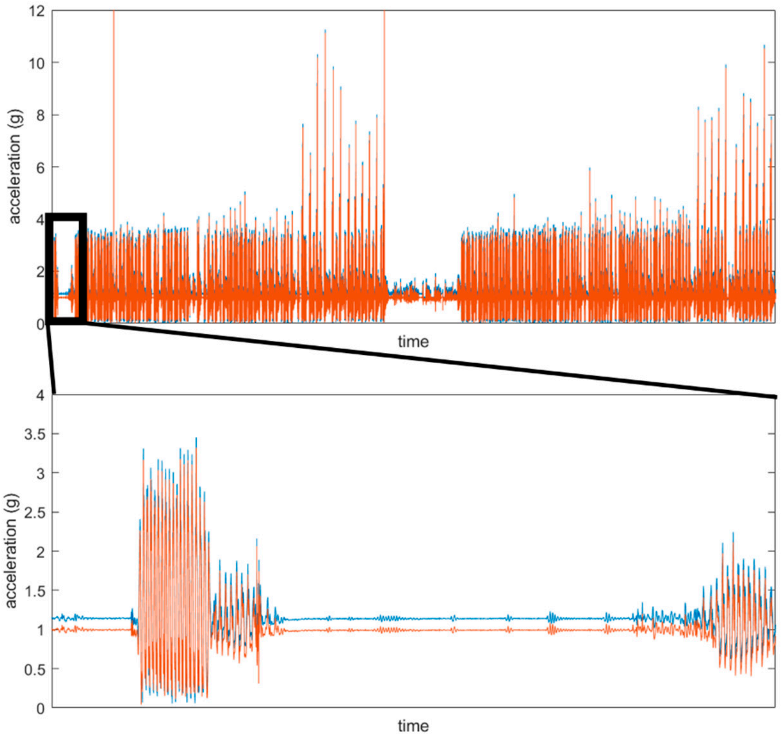

Figure S43. **<u>Top</u>**: Uncorrected resultant sacral acceleration (blue) and de-biased resultant sacral acceleration (orange) across the entire data collection for the randomly selected participant. **<u>Bottom</u>**: Zoomed in to show that uncorrected resultant acceleration does not equal 1 g during quiet periods while de-biased acceleration equals exactly 1 g.

4. *Saturation correction*

Our IMU contained two tri-axial accelerometers with different ranges. The primary accelerometer had a range of 16 g while the secondary accelerometer had a range of 100 g. Although 16 g is a large enough range to capture the majority of accelerations at the tibia and sacrum during running, we wanted to ensure that saturation did not occur, particularly at the tibia [38]. Thus, we used a threshold of 15.5 g and replaced any value above this threshold in our primary accelerometer with the corresponding frame from our secondary accelerometer (these values were highly correlated across the ±16 g range they could both measure). Secondary accelerometer data were then discarded.

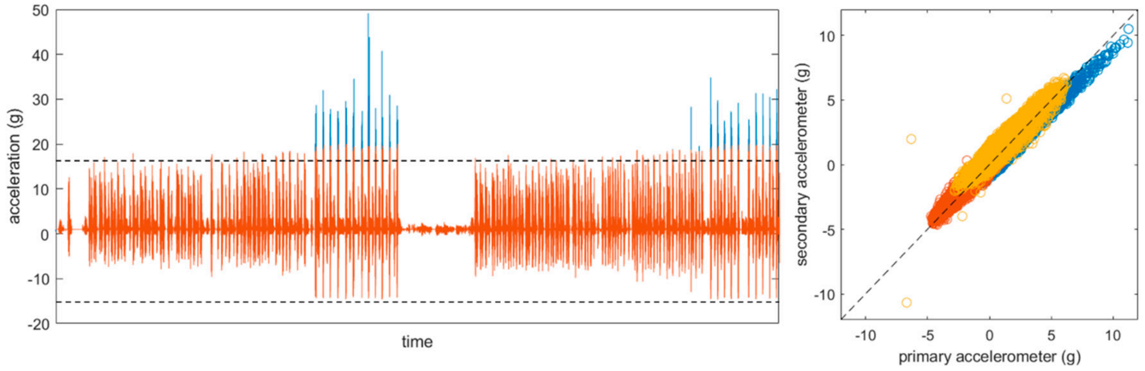

Figure S44. **<u>Left</u>**: Tibia ~longitudinal axis accelerations from primary accelerometer (orange) and secondary accelerometer (blue) across the entire data collection for the example participant. The horizontal black line indicates data outside the primary accelerometer's range (defined as $|a| > 15.5$ g). **<u>Right</u>**: Secondary accelerometer measurements plotted against primary accelerometer measurements for each axis (different colors). Black dashed diagonal line indicates perfect agreement. Here correlations between each axis ranged from r = 0.82 - 0.96. In general, correlations across the ranges shared between primary and secondary accelerometers were ≥ 0.90.

5. *Low pass filtering*

Next, accelerations and angular velocities were filtered with a 4th order 50 Hz low-pass Butterworth filter.

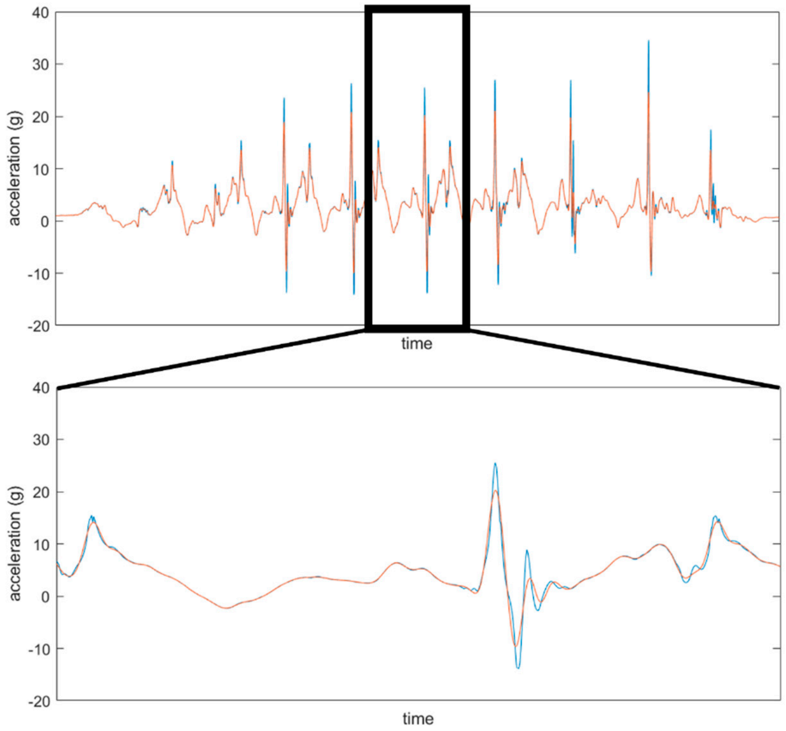

Figure S45. **<u>Top</u>**: Worst-case example of unfiltered (blue) and filtered (orange) ~longitudinal tibial acceleration (that experienced saturation and is now composed of data from the primary and secondary accelerometers). Data is taken from a single trial from the example participant. **<u>Bottom</u>**: Zoomed in on ~one step to better visualize differences between filtered and unfiltered signal. Filter parameters were chosen to qualitatively balance the preservation of major signal features (particularly peak magnitudes and locations) with the removal of high-frequency noise.

6. *Drift correction*

Angular velocity measured by IMUs is prone to drift. This drift makes it difficult to integrate angular velocities and calculate the orientation of an IMU in space. Several sensor fusion algorithms have been developed to correct this drift including Kalman filters [39], Mahoney filters [40], and Madgwick filters [41]. We explored the use of each of these filters and found that converting our data to quaternion representation and entering it into a Madgwick filter (with beta set to 0.05 and no magnetometer fusion due to the amount of magnetic interference in our lab) was the most successful in eliminating drift in a "worst case" recreation of our experimental conditions (an 80 minute data capture with extreme angular rotations and accelerations and no quiet period corrections yielded 1.66 rad rotation error). The code we used to execute the Madgwick filter is available from x-io at:

https://x-io.co.uk/open-source-imu-and-ahrs-algorithms/

And from MATLAB at:

https://www.mathworks.com/products/sensor-fusion-and-tracking.html

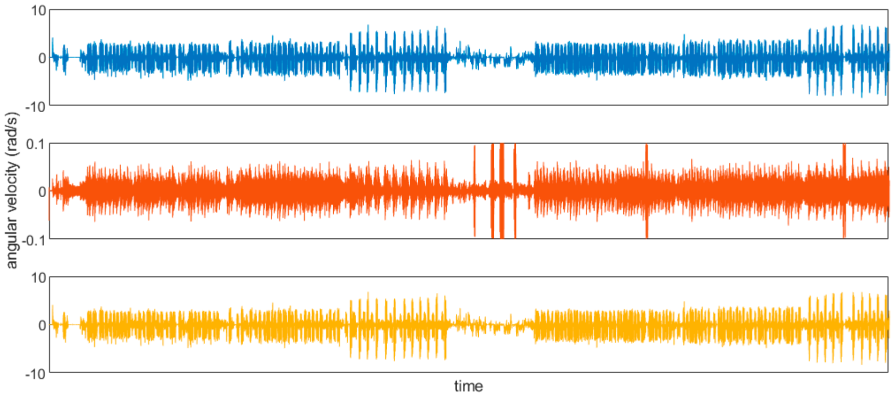

Figure S46. **Top**: Uncorrected angular velocity about the ~longitudinal axis of the sacrum (blue) for the entire data collection of the example participant. **Middle**: Difference between uncorrected and Madgwick filter-estimated angular velocities (orange). **Bottom**: Madgwick filter-estimated angular velocity about the ~longitudinal axis of the sacrum (yellow)

7. *Orientation estimation and gravity subtraction*

After drift-correcting angular velocity with the Madgwick algorithm, we create a rotation matrix based on the loading of gravity during quiet periods (see 3 above) then used it to create a "tilt-corrected" coordinate system (see 8 below). Then, between each quiet period, we used angular velocity to calculate changes in orientation based on Equations 2 and 3 in McGinnis & Perkins [42]. This provided a rotation matrix from the wearable coordinate system to the "tilt-corrected" coordinate system for each time step.

Using these time-varying rotation matrices, acceleration data for each frame was expressed in the "tilt-corrected" coordinate system then 1 g was subtracted from the y-axis (in line with gravity). This procedure removed the gravity component from the accelerometer data. To create the wearable and segment coordinate systems (in the next step), data were then re-expressed in their original coordinate system using the inverse of the time-varying rotation matrices.

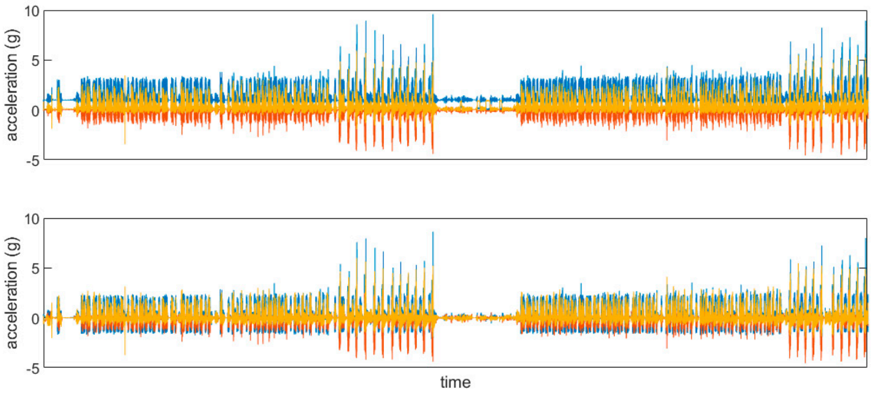

Figure S47. **<u>Top</u>**: Uncorrected acceleration of the sacrum for the entire data collection of the example participant (colors represent different axes). **<u>Bottom</u>**: Acceleration of the sacrum after subtracting 1 from the y-axis in the "tilt-corrected" coordinate system and then re-expressing in the wearable coordinate system (colors represent different axes).

8. *Coordinate system definition*

Finally, data were expressed in three different coordinate systems for analysis. First, data were expressed in the Wearable Coordinate System (WCS). This is *not* the raw coordinate system of the IMU. Rather, all data were corrected with the calibration matrices described in 1 above. These calibration matrices ensured that data were expressed in orthogonal axes aligned with the IMU housing. The IMU housing was positioned such that, during quiet standing, the WCS axes were oriented roughly in the direction of progression (+x), the longitudinal axis (+y), and to the right (+z).

Data were also expressed in a Segment Coordinate System (SCS). This coordinate system was defined using an approach described in the Supplemental Material of Cain et al. [43] which can be found at:

http://dx.doi.org/10.1016/j.gaitpost.2015.10. 022

In brief, accelerations during a quiet standing trial were used to define a gravity vector (similar to 3 above) assuming that the segment was aligned with gravity during the standing trial. This gravity-based vector was defined as the proximal-distal axis (+y proximal). Then a period of steady-state running was manually selected from the data set. Angular velocities from this period were entered into a Principal Component Analysis and the Principal Component accounting for the most variability in angular velocity was selected to represent the average axis of rotation. During running the average axis of rotation was assumed to correspond to the medial-lateral axis. We defined this as the z axis (+z right). The anterior-posterior axis was then defined as the cross-product of y and z (+x anterior). Finally, the z axis was recalculated as the cross-product of x and y to ensure orthogonality. These three unit vectors were then used to create a rotation matrix that transformed data from the WCS to the SCS.

Finally, data were expressed in a pseudo-global system similar to Cain et al.'s "tilt-corrected" coordinate system (TCCS) [43]. First, we created a rotation matrix based on the loading of gravity during quiet periods (see 3 above). This rotation matrix expressed data with the y-axis aligned with gravity during quiet standing (+y vertical). Next, the acceleration of each axis was double integrated to obtain displacement then entered into a Principal Component Analysis. The Principal Component accounting for the most variation in displacement was taken as the projection of the direction of progression onto the horizontal plane (+x direction of progression). Then the projection of the medial-lateral axis onto the horizontal plane was defined as the cross product of x and z (+z right). The

x-axis was then recalculated to ensure orthogonality. These three unit vectors were then used to create a rotation matrix and multiplied by the time-varying rotation matrices described in 7 to express data in the TCCS. Thus, TCCS data is always expressed with y aligned with gravity but with x and z free to rotate about y as the participant moves.

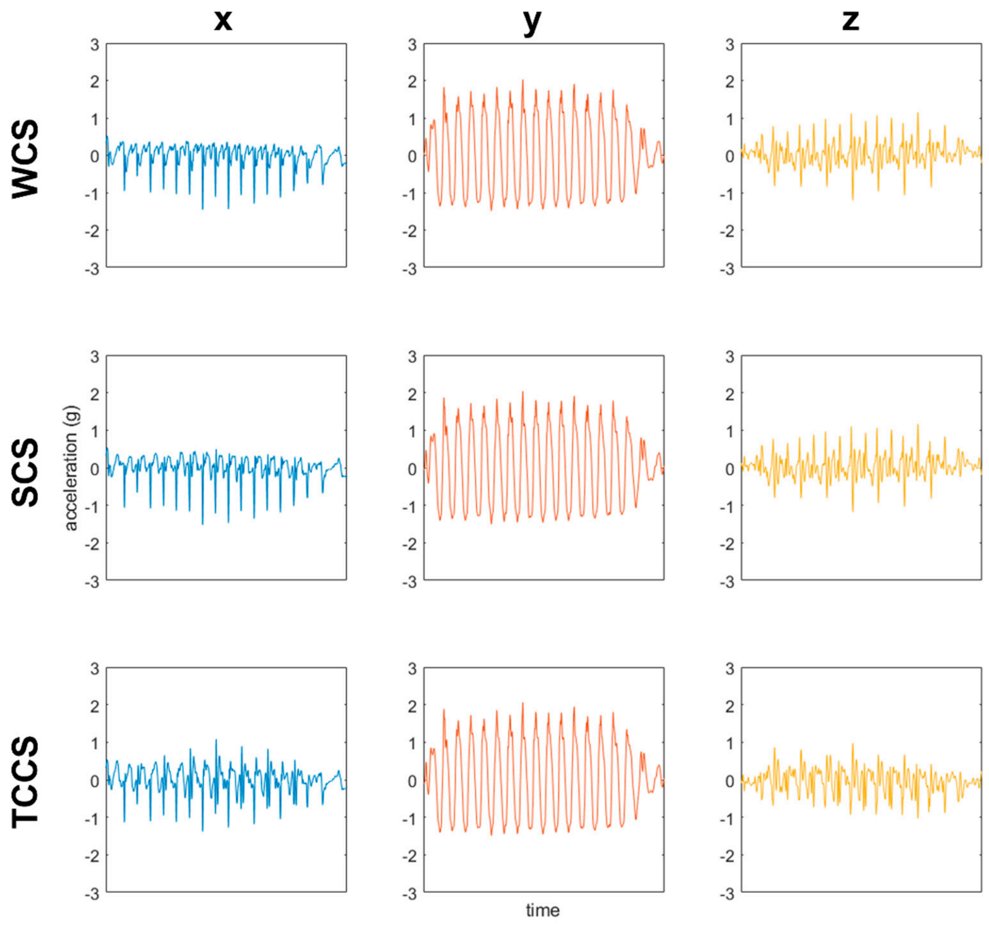

Figure S48. All plots show sacral accelerations from the same trial from the example participant. The **top row** shows data in the Wearable Coordinate System (WCS). the **middle row** shows data in the Segment Coordinate System. The **bottom row** shows data in the Tilt-Corrected Coordinate System (TCCS). The **first column** shows x-axis data (blue). The **second column** shows y-axis data (orange). The **third column** shows z-axis data (yellow). Due to the similarities between all three coordinate systems at the sacrum, discrepancies are minor.

References

1. Cavanagh, P.R.; Lafortune, M.A. Ground reaction forces in distance running. *J. Biomech.* **1980**, *13*, 397–406. https://doi.org/10.1016/0021-9290(80)90033-0.
2. Munro, C.F.; Miller, D.I.; Fuglevand, A.J. Ground reaction forces in running: A reexamination. *J. Biomech.* **1987**, *20*, 147–155. https://doi.org/10.1016/0021-9290(87)90306-x.
3. Cavanagh, P.R.; Kram, R. Stride length in distance running: velocity, body dimesnions, and added mass effects. *Med. Sci. Sport. Exerc.* **1989**, *21*, 467–479.
4. Williams, K.R.; Snow, R.; Arguss, C. Changes in distance running kinematicswith fatigue. *Int. J. Sport Biomech.* **1991**, *7*, 138–162.
5. De Wit, B.; De Clercq, D.; Aerts, P. Biomechanical analysis of the stance phase during barefoot and shod running. *J. Biomech.* **1999**, *33*, 269–278. https://doi.org/10.1016/s0021-9290(99)00192-x.
6. Weyand, P.G.; Sternlight, D.B.; Bellizzi, M.J.; Wright, S. Faster top running sppeds are achieved with greater ground forces not more rapid leg movements. *J. Appl. Physiol.* **2000**, *89*, 1991–1999.
7. Leskinen, A.; Häkkinen, K.; Virmavirta, M.; Isolehto, J.; Kyröläinen, H. Comparison of running kinematics between elite and national-standard 1500-m runners. *Sport. Biomech.* **2009**, *8*, 1–9. https://doi.org/10.1080/14763140802632382.
8. Weyand, P.G.; Sandell, R.F.; Prime, D.N.L.; Bundle, M.W. The biological limits to running speed are imposed from the ground up. *J. Appl. Physiol.* **2010**, *108*, 950–961. https://doi.org/10.1152/japplphysiol.00947.2009.
9. Meardon, S.A.; Hamill, J.; Derrick, T.R. Running injury and stride time variability over a prolonged run. *Gait Posture* **2011**, *33*, 36–40. https://doi.org/10.1016/j.gaitpost.2010.09.020.
10. Mizrahi, J.; Verbitsky, O.; Isakov, E.; Daily, D. Effect of fatigue on leg kinematics and impact acceleration in long distance running. *Hum. Mov. Sci.* **2000**, *19*, 139–151. https://doi.org/10.1016/s0167-9457(00)00013-0.
11. Mercer, A.J.; Bates, B.T.; Dufek, J.; Hreljac, A. Characteristics of shock attenuation during fatigued running. *J. Sport. Sci.* **2003**, *21*, 911–919. https://doi.org/10.1080/0264041031000140383.
12. Shorten, M.R.; Winslow, D.S. Spectral Analysis of Impact Shock during Running. *Int. J. Sport. Biomech.* **1992**, *8*, 288–304. https://doi.org/10.1123/ijsb.8.4.288.
13. Hamill, J.; Derrick, T.; Holt, K. Shock attenuation and stride frequency during running. *Hum. Mov. Sci.* **1995**, *14*, 45–60. https://doi.org/10.1016/0167-9457(95)00004-c.
14. Derrick, T.R.; Hamill, J.; Caldwell, G.E. Energy absorption of impacts during running at various stride lengths. *Med. Sci. Sport. Exerc.* **1998**, *30*, 128–135. https://doi.org/10.1097/00005768-199801000-00018.
15. Derrick, T.R.; Dereu, D.; McLean, S.P. Impacts and kinematic adjustments during an exhaustive run. *Med. Sci. Sport. Exerc.* **2002**, *34*, 998–1002. https://doi.org/10.1097/00005768-200206000-00015.
16. Purcell, B.; Channells, J.; James, D.; Barrett, R. Use of accelerometers for detecting foot-ground contact time during running. **2005**, *6036*, 292–299. https://doi.org/10.1117/12.638389.
17. Aminian, K.; Najafi, B.; Büla, C.; Leyvraz, P.-F.; Robert, P. Spatio-temporal parameters of gait measured by an ambulatory system using miniature gyroscopes. *J. Biomech.* **2002**, *35*, 689–699. https://doi.org/10.1016/s0021-9290(02)00008-8.
18. O'Donovan, K.; Greene, B.; McGrath, D.; O'Neill, R.; Burns, A.; Caulfield, B. SHIMMER: A new tool for temporal Gait analysis. In Proceedings of the 31st Annual International Conference of the IEEE EMBS, Minneapolis, MN, USA, 3–6 September 2009.
19. Greene, B.R.; McGrath, D.; O'neill, R.; O'donovan, K.J.; Burns, A.; Caulfield, B. An adaptive gyroscope-based algorithm for temporal gait analysis. *Med. Biol. Eng. Comput.* **2010**, *48*, 1251–1260. https://doi.org/10.1007/s11517-010-0692-0
20. McGrath, D.; Greene, B.R.; O'donovan, K.J.; Caulfield, B. Gyroscope-based assessment of temporal gait parameters during treadmill walking and running. *Sport. Eng.* **2012**, *15*, 207–213. https://doi.org/10.1007/s12283-012-0093-8.
21. Sinclair, J.; Hobbs, S.J.; Protheroe, L.; Edmundson, C.J.; Greenhalgh, A. Determination of Gait Events Using an Externally Mounted Shank Accelerometer. *J. Appl. Biomech.* **2013**, *29*, 118–122. https://doi.org/10.1123/jab.29.1.118.
22. Whelan, N.; Healy, R.; Kenny, I.; Harrison, A. A comparison of foot strike events using the force plate and peak impact acceleration measures. In *International Society of Biomechanics in Sport 33*; Poitiers, France, 2015.
23. Norris, M.; Kenny, I.C.; Anderson, R. Comparison of accelerometry stride time calculation methods. *J. Biomech.* **2016**, *49*, 3031–3034. https://doi.org/10.1016/j.jbiomech.2016.05.029.
24. Schmidt, M.; Rheinländer, C.; Nolte, K.F.; Wille, S.; Wehn, N.; Jaitner, T. IMU- based Determination of Stance Duration during Sprinting. *Procedia Eng.* **2016**, *147*, 747–752. https://doi.org/10.1016/j.proeng.2016.06.330.
25. Aubol, K.G.; Milner, C. Foot contact identification using a single triaxial accelerometer during running. *J. Biomech.* **2020**, *105*, 109768. https://doi.org/10.1016/j.jbiomech.2020.109768.
26. Fadillioglu, C.; Stetter, B.J.; Ringhof, S.; Krafft, F.C.; Sell, S.; Stein, T. Automated gait event detection for a variety of locomotion tasks using a novel gyroscope-based algorithm. *Gait Posture* **2020**, *81*, 102–108. https://doi.org/10.1016/j.gaitpost.2020.06.019.
27. Bach, M.M.; Dominici, N.; Daffertshofer, A. Predicting vertical ground reaction forces from 3D accelerometery using reservoir computers leads to accurate gait event detection. *BioRxiv* **2022**. https://doi.org/10.3389/fspor.2022.1037438.
28. Auvinet, B.; Gloria, E.; Renault, G.; Barrey, E. Runner's stride analysis: comparison of kinematic and kinetic analyses under field conditions. *Sci. Sport.* **2002**, *17*, 92–94. https://doi.org/10.1016/s0765-1597(02)00122-3.
29. Lee, J.; Mellifont, R.; Burkett, B. The use of a single inertial sensor to identify stride, step, and stance durations of running gait. *J. Sci. Med. Sport* **2010**, *13*, 270–273.
30. Wixted, A.; Billing, D.; James, D. Validation of trunk mounted inertial sensors for analysing running biomechanics under field conditions, using synchronously collected foot contact data. *Sport. Eng.* **2010**, *12*, 207–212.

31. Bergamini, E.; Picerno, P.; Pillet, H.; Natta, F.; Thoreux, P.; Camomilla, V. Estimation of temporal parameters during sprint running using a trunk-mounted inertial measurement unit. *J. Biomech.* **2012**, *45*, 1123–1126. https://doi.org/10.1016/j.jbiomech.2011.12.020.
32. Luo, J.; Bai, J.; Shao, J. Application of the wavelet transforms on axial strain calculation in ultrasound elastography. *Prog. Nat. Sci.* **2006**, *16*, 942–947. https://doi.org/10.1080/10020070612330093.
33. Benson, L.; Clermont, C.; Watari, R.; Exley, T.; Ferber, R. Automated accelerometer-based gait event detection during multiple running conditions Lauren. *Sensors* **2019**, *19*, 1483.
34. Reenalda, J.; Zandbergen, M.A.; Harbers, J.H.; Paquette, M.R.; Milner, C.E. Detection of foot contact in treadmill running with inertial and optical measurement systems. *J. Biomech.* **2021**, *121*, 110419. https://doi.org/10.1016/j.jbiomech.2021.110419.
35. United States Geological Survey. Gravity Anamoly Map of the Continental United States. Available online: https://mrdata.usgs.gov/gravity/map-us.html#home (accessed on 5 June 2019).
36. National Geodtic Survey. NGS Surface Gravity Prediction. National Oceanic and Atmospheric Administration. Available online: https://www.ngs.noaa.gov/cgi-bin/grav_pdx.prl (accessed on 5 June 2019).
37. Coolbaugh, C.L.; Hawkins, D.A. Standardizing Accelerometer-Based Activity Monitor Calibration and Output Reporting. *J. Appl. Biomech.* **2014**, *30*, 594–597. https://doi.org/10.1123/jab.2013-0240.
38. Lafortune, M.A. Three-dimensional acceleration of the tibia during walking and running. *J. Biomech.* **1991**, *24*, 877–886. https://doi.org/10.1016/0021-9290(91)90166-k.
39. Kalman, R.E. A New Approach to Linear Filtering and Prediction Problems. *J. Basic. Eng.* **1960**, *82*, 35–45. https://doi.org/10.1115/1.3662552.
40. Mahony, R.; Hamel, T.; Pflimlin, J.-M. Nonlinear Complementary Filters on the Special Orthogonal Group. *IEEE Trans. Autom. Control.* **2008**, *53*, 1203–1218. https://doi.org/10.1109/tac.2008.923738.
41. Madgwick, S. *An Efficient Orientation Filter for Inertial and Inertial/Magnetic Sensor Arrays*; x-io: Bristol, UK, 2010.
42. McGinnis, R.S.; Perkins, N.C. A Highly Miniaturized, Wireless Inertial Measurement Unit for Characterizing the Dynamics of Pitched Baseballs and Softballs. *Sensors* **2012**, *12*, 11933–11945.
43. Cain, S.M.; McGinnis, R.S.; Davidson, S.P.; Vitali, R.V.; Perkins, N.C.; McLean, S.G. Quantifying performance and effects of load carriage during a challenging balancing task using an array of wireless inertial sensors. *Gait Posture* **2016**, *43*, 65–69. https://doi.org/10.1016/j.gaitpost.2015.10.022.
